# π-Stacking Isomerism in Polycyclic Aromatic Hydrocarbons: The 2-Naphthalenethiol Dimer

**DOI:** 10.1021/acs.jpclett.2c03299

**Published:** 2022-12-30

**Authors:** Rizalina Tama Saragi, Camilla Calabrese, Marcos Juanes, Ruth Pinacho, José Emiliano Rubio, Cristóbal Pérez, Alberto Lesarri

**Affiliations:** † Departamento de Química Física y Química Inorgánica, Facultad de Ciencias - I.U. CINQUIMA, Universidad de Valladolid, Paseo de Belén, 7, E-47011 Valladolid, Spain; § Departamento de Electrónica, ETSIT, Universidad de Valladolid, Paseo de Belén, 11, E-47011 Valladolid, Spain

## Abstract

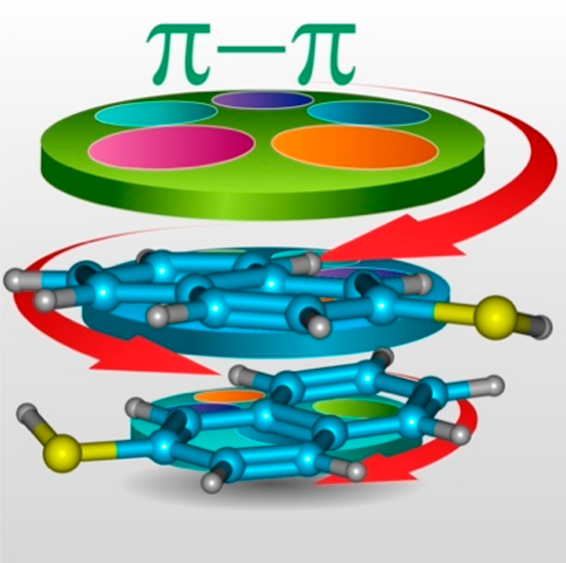

π-Stacking is a common descriptor for face-to-face attractive forces between aromatic hydrocarbons. However, the physical origin of this interaction remains debatable. Here we examined π-stacking in a model homodimer formed by two thiol-substituted naphthalene rings. Two isomers of the 2-naphthalenethiol dimer were discovered using rotational spectroscopy, sharing a parallel-displaced crossed orientation and absence of thiol–thiol hydrogen bonds. One of the isomers presents *C*_2_ symmetry, structurally analogous to the global minimum of the naphthalene dimer. The experimental data were rationalized with molecular orbital calculations, revealing a shallow potential energy surface. Noncovalent interactions are dominated by dispersion forces according to SAPT energy decomposition. In addition, the reduced electronic density shows a diffuse and extended region of inter-ring interactions, compatible with the description of π-stacking as a competition between dispersion and Pauli repulsion forces.

π-Stacking interactions are ubiquitous in chemical^1^ and biological systems^2,3^ and constitute a valuable tool in the engineering of supramolecular assemblies,^4,5^ crystal designs,^6^ host–guest compounds,^7^ catalysis,^8^ and materials.^9^ At the same time, π-stacking is considered one of the most controversial supramolecular interactions,^10,11^ and most of present information is still essentially descriptive. Electronic computations can establish the main features of the potential energy surface (PES), but they miss a clear understanding of the physical origin of stacking.^12^ Moreover, nearly all empirical data arise from crystal diffraction,^4,13^ and there is a lack of gas-phase experiments which could provide benchmarking structural evidence unbiased from matrix effects.

The ideas on π-stacking have evolved considerably. The Hunter–Sanders model attributed the observed geometries to a balance between (classical) quadrupolar electrostatics and London dispersion interactions.^14^ This model has largely influenced supramolecular chemistry^5^ but fails for some simple systems like benzene-hexafluorobenzene.^12^ Sherrill^15−17^ and Wheeler^18,19^ have criticized several aspects of this model, in particular the need of quantum electrostatics and the importance of penetration effects at the shorter distances of π–π or CH···π interactions. Grimme analyzed larger arene dimers, emphasizing the prevailing role of dispersion forces.^10^ More recently, Carter-Fenk and Herbert^12^ offered a radically different paradigm, in which electrostatics is sidelined and dispersion and Pauli repulsion constitute the main contributors to π-stacking. As computational studies become more complex, there is a recurrent need for contrasting experiments on π-bonded systems. Among gas-phase techniques, jet-cooled vibrational^20−24^ and rotational^25−31^ spectroscopies provide cluster generation and characterization of weakly bound dimers, offering high-resolution structural information directly comparable to theory. The number of gas-phase studies of π-stacking polycyclic aromatic hydrocarbon (PAH) dimers is nevertheless quite small.

In the flat and deceptively simple PES of the prototype benzene dimer, both the perpendicular (T-shape) and parallel (cofacial) canonical forms are saddle points,^32−34^ and the lowest-lying minima correspond to the parallel-displaced and the tilted-T-shape geometry, which was experimentally detected in the gas phase using rotational spectroscopy.^26−28^ Unsurprisingly, the spectrum confirmed that the weakly bound benzene dimer exhibits notorious internal dynamical effects,^28^ quite difficult to reproduce theoretically and impossible to observe in the crystal. We recently observed also large-amplitude motions in the rotational spectrum of the thiophenol dimer, which, unlike the hinged phenol dimer,^35^ combine a parallel-displaced geometry with a weak S–H···S hydrogen bond.^30^ A parallel-displaced geometry and torsional tunnellings were similarly observed for the *o*-difluorobenzene dimer.^25^ For bicyclic aromatic hydrocarbons, the naphthalene dimer may adopt four possible geometries, the global minimum being a stacked geometry with the two rings in a crossed V-shape geometry.^36,37^ However, this dimer could only be detected vibrationally because of its small dipole moment,^24^ and rotational observations have been limited to the dimer of 1-naphthol^29^ and the tricyclics of fluorene and dibenzofuran.^31^ In order to understand why parallel-displaced geometries are the dominant structures for larger PAHs,^38^ including biological compounds^2^ and heterocycles,^39,40^ other arene dimers should be studied in the gas phase.

Here we report a rotational investigation on the dimer of 2-naphthalenethiol using broadband microwave spectroscopy,^41^ density-functional theory (B3LYP, ωB97XD, and B2PLYP), and ab initio (SCS-MP2 and DLPNO-CCSD(T)) calculations. The rationale for this study was the introduction of a dipole moment in the molecule through the thiol polar bond, simultaneously offering comparison with the dimers of naphthalene^36^ and 1-naphthol,^29^ gauging substituent effects^15,18,42,43^ and comparing plausible hydrogen bonds^30,44^ originated by the thiol group. The results constitute the first rotational detection of π-stacking isomerism in bicyclic aromatic hydrocarbons, offering insight on the PES and the structural and electronic properties of these weak noncovalent interactions.

The monomer of 2-naphthalenethiol adopts two planar *cis* or *trans* conformations, depending on the orientation of the thiol group. *cis*-2-Naphthalenethiol is the global minimum but quite similar in energy to the *trans* conformer (B3LYP-D3(BJ): 0.6 kJ mol^–1^ in Figure S1). For the 2-naphthalenethiol dimer, 47 *cis*/*cis* (CC), *cis*/*trans* (CT), or *trans*/*trans* (TT) structures were investigated computationally (see Supporting Information). *A priori*, the dimer structure may use sandwich, parallel-displaced, or T-shaped geometries similar to the naphthalene,^36^ benzene,^32−34^ or the thiophenol^30^ dimers, but hydrogen-bonded hinged geometries similar to the phenol dimer^35^ were also considered. However, neither T-shaped nor hinged geometries converged to stable dimer structures, and all predicted isomers displayed parallel-displaced stacked geometries. The calculated PES was quite flat, and the 19 most stable isomers of Figure S2 are distributed within a small energy window of only 4.3 kJ mol^–1^ (B3LYP). The five lowest-lying isomers in Figures 1 and S3 differ by less than 0.9 kJ mol^–1^ (B3LYP) and are characterized by the absence of any S–H···S hydrogen bond (predicted at electronic energies above 1.1 kJ mol^–1^). The parallel-displaced forms may present different geometries depending on the orientation between the two naphthalene subunits, occasionally adopting symmetric structures like the crossed or slipped geometries, as in the *C*_2_ global minimum (CC-1). The results of the model calculations are compared in Tables S1–S3 (B3LYP), S4 (ωB97XD), S5 (B2PLYP), S6 (RI-SCS-MP2), and S7 (DLPNO-CCSD(T)). B3LYP and B2PLYP predict CC-1 as global minimum, but the prediction is reversed for ωB97XD and the ab initio methods, which favor CC-2.

**Figure 1:**
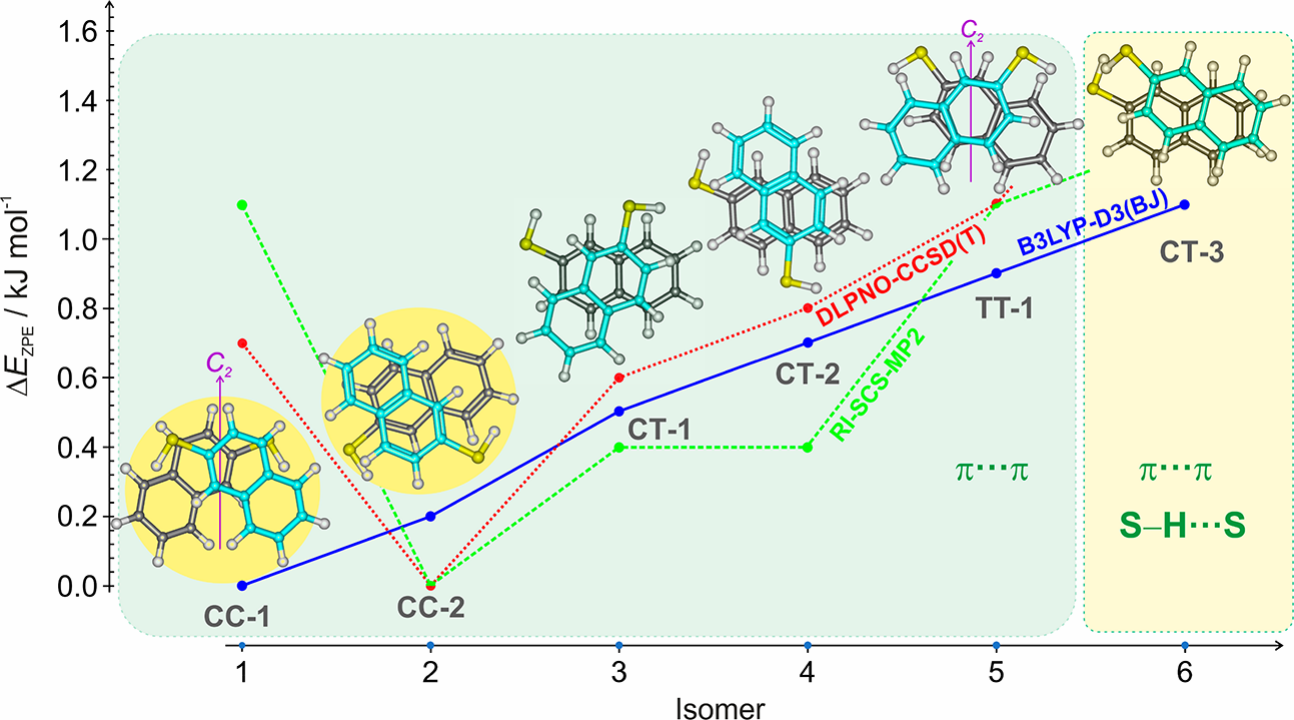
Two detected species and partial PES of the 2-naphthalenethiol dimer (see also Tables S1–S7 and Figure S3). The two *cis*-*cis* (CC-1 and CC-2) isomers observed experimentally are highlighted on the left. The conformational search used initially a DFT method (B3LYP-D3(BJ), blue trace) and the most stable isomers were later reoptimized at different calculation levels (the RI-SCS-MP2 and DLPNO-CCSD(T) results in green and red do not include zero-point corrections). The most stable isomers lack any S–H···S hydrogen bond. In all cases, stacked structures are preferred.

The molecular-jet microwave spectrum of Figure S4 was then surveyed for the signals of the 2-naphthalenethiol dimer, expected to peak in the 2–8 GHz region at the effective rotational temperature of ca. 2 K. The spectral analysis is discussed in the Supporting Information. Two weak spectral signatures were detected, confirming the competition between two isomers of the dimer. The experimental data set, comprising more than 250 transitions, is presented in Tables S8 and S9. The derived experimental parameters are collected in Table 1. Isotopic species in natural abundance were undetectable. No other dimer species were identified.

**Table 1:** Rotational Parameters for the 2-Naphthalenethiol Dimer and Computational Predictions

	Experiment		Theory					
	Isomer 1	Isomer 2	CC-1	CC-2	CT-1	CT-2	TT-1	CT-3
*A*/MHz^a^	308.38853(21)^e^	299.45856(51)	318.10	304.23	294.60	307.2	300.3	390.1
*B*/MHz	231.75029(16)	246.9652(12)	233.40	250.65	245.68	249.6	242.1	228.2
*C*/MHz	226.78483(18)	221.5793(15)	231.34	225.72	240.89	225.6	239.4	175.3
*D*_*J*_/kHz	0.01761(44)	0.1328(56)	0.016	0.012	0.024	0.012	0.022	0.010
*D*_*JK*_/kHz	[0.0]^f^	–0.242(14)	–0.003	0.028	–0.037	0.032	–0.024	0.005
*D*_*K*_/kHz	0.0234(12)	0.164(10)	0.023	0.005	0.055	0.001	0.044	0.065
*d*_1_/kHz	[0.0]	0.0434(28)	–0.002	0.000	–0.007	0.000	–0.009	–0.001
*d*_2_/kHz	[0.0]	[0.0]	0.001	–0.001	0.002	–0.001	0.001	–0.001
|μ_*a*_|/D	-	-	0.0	0.2	1.0	–0.7	0.0	–1.1
|μ_*b*_|/D	-	++	0.0	0.6	1.1	1.3	0.0	1.2
|μ_*c*_|/D	+++^g^	++	1.3	0.6	0.6	1.2	–2.1	0.0
*N*^b^	173	86						
σ/kHz	10.6	10.6						
Δ*E*_ZPE_/kJ mol^–1^^c^			0.0	0.2	0.5	0.7	0.9	1.1
Δ*G*/kJ mol^–1^			1.4	1.8	0.0	0.2	–1.1	1.3
*E*_C_/kJ mol^–1^			–48.7	–47.4	–47.2	–47.2	–46.9	–46.9
Δ*E*_SCS-MP2_/kJ mol^–1^^d^			1.1	0.0	0.4	0.4	1.1	1.3
Δ*E*_DLPNO-CCSD(T)_/kJ mol^–1^			0.7	0.0	0.6	0.8	1.1	2.4

a Rotational constants (*A*, *B*, *C*), centrifugal distortion constants (*D*_*J*_, *D*_*JK*_, *D*_*K*_, *d*_1_, *d*_2_) according to Watson’s S-reduction (I^r^-representation) and electric dipole moments (μ_α_, α = *a*, *b*, *c*).

b Number of measured transitions (*N*) and standard deviation of the fit (σ).

c Relative energy with zero-point corrections (Δ*E*), Gibbs energy (Δ*G*, 298 K, 1 atm), and complexation energy (Δ*E*_C_ including BSSE corrections) using B3LYP-D3(BJ)/def2-TZVP.

d Electronic energy using SCS-MP2 and DLPNO-CCSD(T), uncorrected for zero-point vibrational energy.

e Standard errors in parentheses in units of the last digit.

f Parameters in square brackets fixed to zero.

g The plus signs denote qualitatively the observation of the corresponding rotational transitions.

The spectroscopic parameters allowed for an unequivocal isomer identification. In particular, the presence of a symmetry axis matches the predictions for CC-1. Rotatable 3D figures and coordinates for both isomers are shown in Figures S5–S6 and Tables S10–S11. The experiment–theory comparison in Table 1 gives a good structural agreement for the B3LYP-D3(BJ) model, with relative differences in the rotational constants of 0.7–3.1% for isomer CC-1 and 1.5–1.9% for isomer CC-2. A comparison with the alternative theoretical models is shown in Table S12 (SI). Interestingly, neither ωB97XD nor the double-hybrid B2PLYP offered significant improvements over B3LYP in structural terms. The basis set dependence on B3LYP is shown in Table S13. The size of the system prevented the calculation of vibrational frequencies and zero-point energies for the B2PLYP, RI-SCS-MP2, and DLPNO-CCSD(T) models.

The noncovalent interactions (NCIs in the 2-naphthalenethiol dimers have been analyzed using structural, energetic, and electronic information. Despite only a few bicyclic aromatic hydrocarbon dimers having been detected so far in the gas phase, some structural patterns now become apparent. Noticeably, hydrogen-bonded structures are not dominant after insertion of an alcohol or thiol group in the naphthalene dimer, preserving the stacked geometry. In the naphthalene dimer, the global minimum shows the parallel-displaced crossed *C*_2_-symmetric structure of Figure 2.^36,37^ In this structure, the largest ring overlap occurs between two terminal rings, favoring the torsioned symmetric arrangement. This *C*_2_ structure was now observed for the 2-naphthalenethiol dimer CC-1, but not in 1-naphthol,^29^ where it is predicted higher in energy (1.0–1.6 kJ mol^–1^). However, the weak balance of intermolecular forces results in different inter-ring torsion angles, much larger in naphthalene^36,37^ (MP2: 135–136°) than for the CC-1 structure of 2-naphthalenethiol (B3LYP: 81°, Figure S7). A second type of limit dimer structure corresponds to ring overlapping between one of the aromatic rings and the central region of the second naphthalene molecule, rotated ca. 66° (*D*_2*d*_ in the naphthalene dimer, Figure S8). This geometry is the global minimum of the 1-naphthol dimer, where it was denoted as V-shape.^29^ We also observed this second structure in the CC-2 dimer, nearly isoenergetic to (B3LYP: +0.2 kJ mol^–1^) or more stable than CC-1 for the ωB97XD (−0.7 kJ mol^–1^) and the ab initio models (SCS-MP2: −1.1 kJ mol^–1^; DLPNO-CCSD(T): −0.7 kJ mol^–1^).

**Figure 2:**
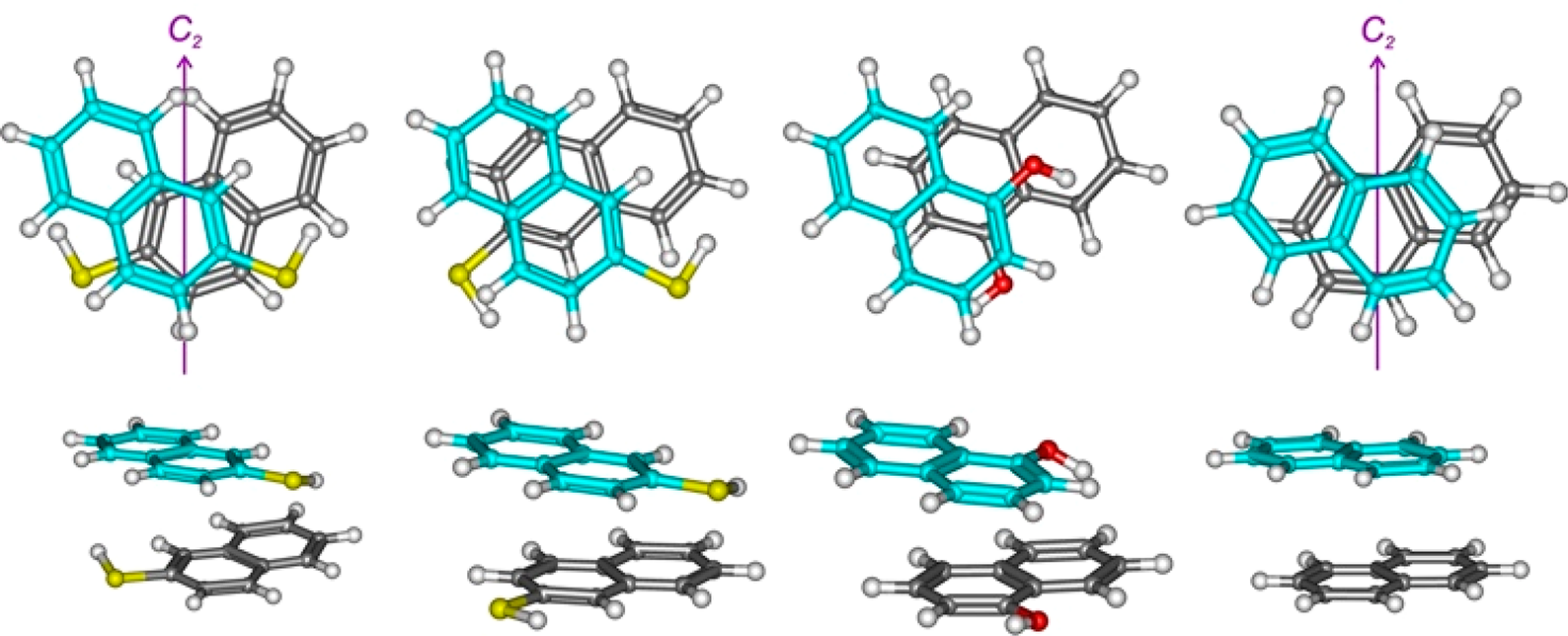
Comparison of the two observed structures of the 2-naphthalenethiol dimer (left) with the dimers of 1-naphthol (center) and naphthalene (right).

In both the 2-naphthalenethiol and 1-naphthol dimers, the polar bond preferentially adopts the monomer conformation (i.e., CC in the first case, TT in the second). However, CT and TT isomers generate competing isomers in the thiol. As an example, isomer TT-1 (B3LYP: 0.9 kJ mol^–1^) is structurally analogous to CC-1. The eventual presence of an S–H···S hydrogen bond is slightly destabilized (B3LYP: >1.1 kJ mol^–1^) but does not affect the global stacking arrangements, with thiol–thiol contacts of *r*(S–H···S) = 2.86–2.94 Å in Figure S9. Hinged structures are energetically excluded for the thiol or alcohol dimers, but they are much lower in energy for the stronger O–H···O hydrogen bond (>1.8 kJ mol^–1^). The weaker interaction associated to the thiol group is also reflected in the flatter and more corrugated PES of this dimer. The inter-ring distances of the most stable dimers (3.26–3.29 Å), also in Figure S9, are similar to those predicted for the naphthalene dimer, exposing the common origin of the attractive stacking interaction.

Despite the observed structures suggesting apparently small substituent effects, the complexation energies for the two 2-naphthalenethiol dimers in Tables 1 and S1–S5 range between −48.7 and 47.3 kJ mol^–1^ using B3LYP or B2PLYP. This value is much larger than the energies of −42.2 and −31.0 kJ mol^–1^ (B3LYP) for the 1-naphthol and naphthalene dimers, exposing the important stabilizer role associated to the presence of the polar bonds. The ωB97XD complexation energies of Table S4 are ca. 3 kJ mol^–1^ smaller. The experimental detection of two isomers suggests interconversion barriers exceeding the collisional relaxation thresholds in the jet (5–12 kJ mol^–1^).^45^ However, the weak character of the 2-napthalenethiol dimer and the multiplicity of conversion paths made the barrier calculation difficult and very sensitive to the calculation method. The GRRM-IRC calculations in Figure S10 estimated a barrier height above 14 kJ mol^–1^ for isomer interconversion, involving a complex multistep route of three different intermediates at B3LYP-D3(BJ)/def2-TZVP level. However, considering the tiny conformational differences, we do not exclude other classes of barriers due to sliding/reorientation, SH internal rotation, and naphthalene face-to-face flipping.

Noncovalent interactions were mapped using Johnson–Contreras’s reduced gradient  of the electronic density (ρ).^46^ The NCI plot in Figure 3 reveals a wide region of weak interactions between the two rings with small pockets of more attractive forces, characteristic of the extended spatial distribution of stacking interactions. The uneven distribution of attractive forces was interpreted in the 1-naphthol dimer as a competition between attractive interactions and Pauli repulsion.^29^ Comparison of the plot of the reduced density gradient vs the signed density for the naphthalene or 1-naphthol dimers also in Figure 3 shows similar patterns. In particular, the negative minimum associated to the most attractive interaction is quite diffuse, and the signatures of the S–H···H or O–H···O hydrogen bonds, observed in the phenol and thiophenol dimers of Figure S11, are absent here. This representation confirms the weak nature of the π-stacking interactions in these dimers. Other interactions are difficult to ascertain. A small tilt of the terminal thiol hydrogens may be indicative of weak cooperative S–H···π interactions, as suggested for the O–H···π contacts in the 1-naphthol dimer, previously categorized as van der Waals forces.^29^

**Figure 3:**
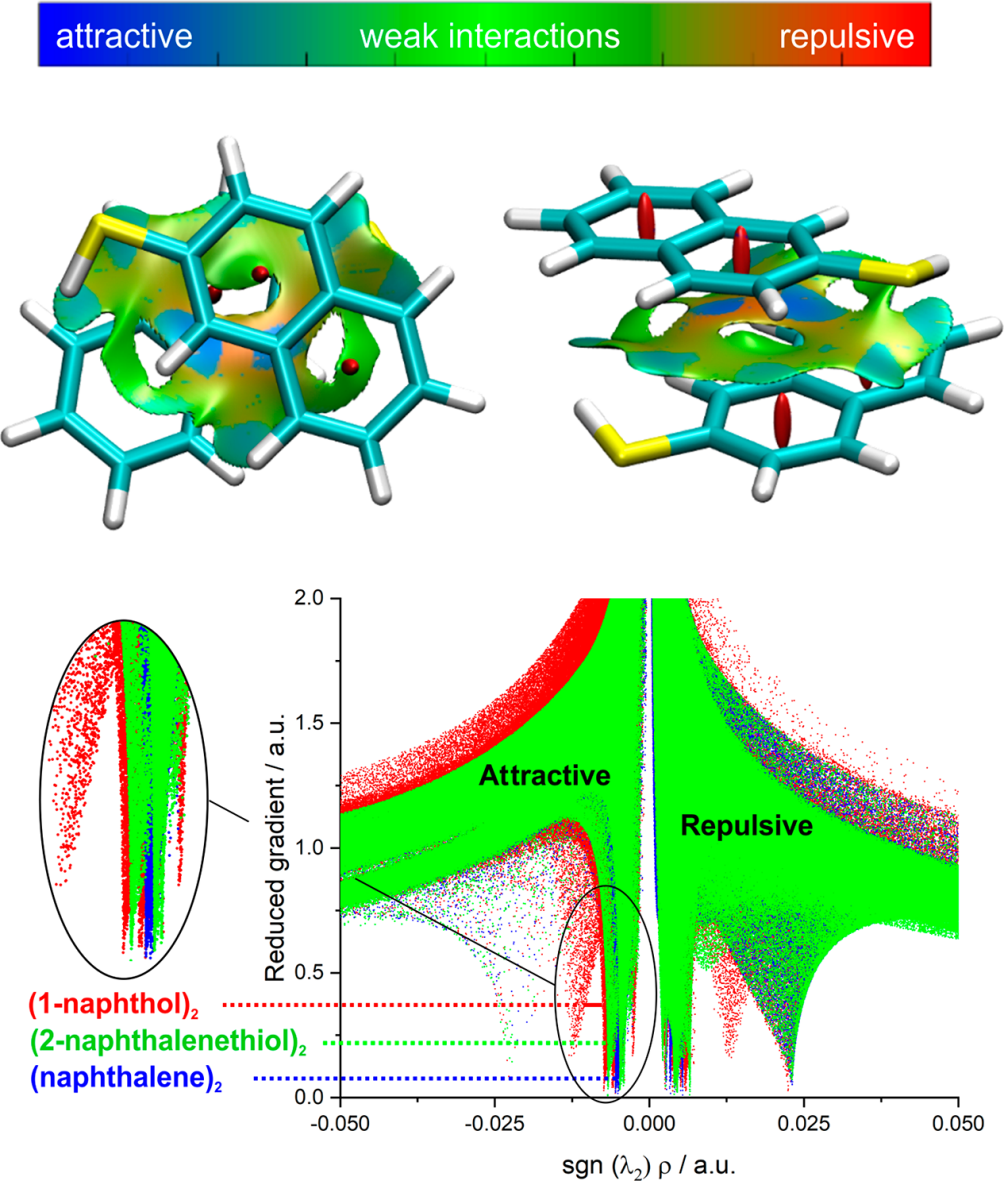
NCI plots (upper panel, isovalue *s* = 0.5) mapping the weak attractive interactions for the CC-1 isomer of the 2-naphthalenethiol dimer, together with a representation of the reduced gradient vs the signed electronic density in the dimers of 2-naphthalenethiol, 1-naphthol, and naphthalene (lower panel, see also Figure S11).

We examined in Table 2 the balance of electrostatic and dispersion forces in the dimer stability, using energy decomposition at SAPT2+(3) level. As expected, the 2-naphthalenethiol is dominated by dispersion forces, which account for 66% of the total attractive interactions compared to a 27% of electrostatic contributions. This energetic composition is quite similar to the naphthalene dimer and other previously observed mono and bicyclic homodimers displaying π-stacking structures, like thiophenol or 1-naphthol. Actually, the representations in Figures 4 and S12 show nearly disjoint regions when compared to prototype hydrogen-bonded dimers using either O–H···O or S–H···H contacts. This dichotomy is especially evident for the dimers of thiophenol and phenol, which differ only in the heteroatom. The different nature of the π-stacking regime and hydrogen-bonded interactions is thus well captured by the combination of NCI plots and SAPT calculation.

**Table 2:** Results from a Binding Energy Decomposition Using (Second-Order Intramonomer/Third-Order Intermonomer) Symmetry-Adapted Perturbation Theory (SAPT2+(3)/aug-cc-pVDZ) for the π-Stacking Homodimers of 2-Naphthalenethiol, Naphthalene, 1-Naphthol, and Thiophenol and Several Hydrogen-Bonded Dimers, Comparing the Magnitude of the Attractive Contributions and Stabilization Energies^a^

	Δ*E*_Electrostatic_	Δ*E*_Induction_	Δ*E*_Dispersion_	Δ*E*_Exchange_	Δ*E*_Total_
(H_2_S)_2_^b^	–12.1 [49.0%]^c^	–4.8 [19.4%]	–7.8 [31.6%]	19.3	–5.4
(H_2_O)_2_	–35.7 [63.4%]	–11.1 [19.7%]	–9.5 [16.9%]	37.7	–18.6
(Phenol)_2_	–41.8 [48.3%]	–15.9 [18.4%]	–28.8 [33.3%]	58.9	–27.6
(1-Naphthol)_2_	–33.4 [27.5%]	–11.0 [9.1%]	–77.1 [63.5%]	80.2	–41.3
(Naphthalene)_2_	–20.5 [21.4%]	–7.0 [7.3%]	–68.1 [71.2%]	65.2	–30.3
(2-Naphthalenethiol)_2_	–34.1 [26.7%]	–8.8 [6.9%]	–84.7 [66.4%]	80.1	–47.5
(Thiophenol)_2_	–26.2 [29.8%]	–8.4 [9.6%]	–53.3 [60.6%]	61.0	–26.9

a All values in kJ mol^–1^.

b The dimer structures were optimized at B3LYP-D3(BJ)/def2-TZVP level.

c The values in square brackets represent the relative percentage with respect to the total attractive interactions.

**Figure 4:**
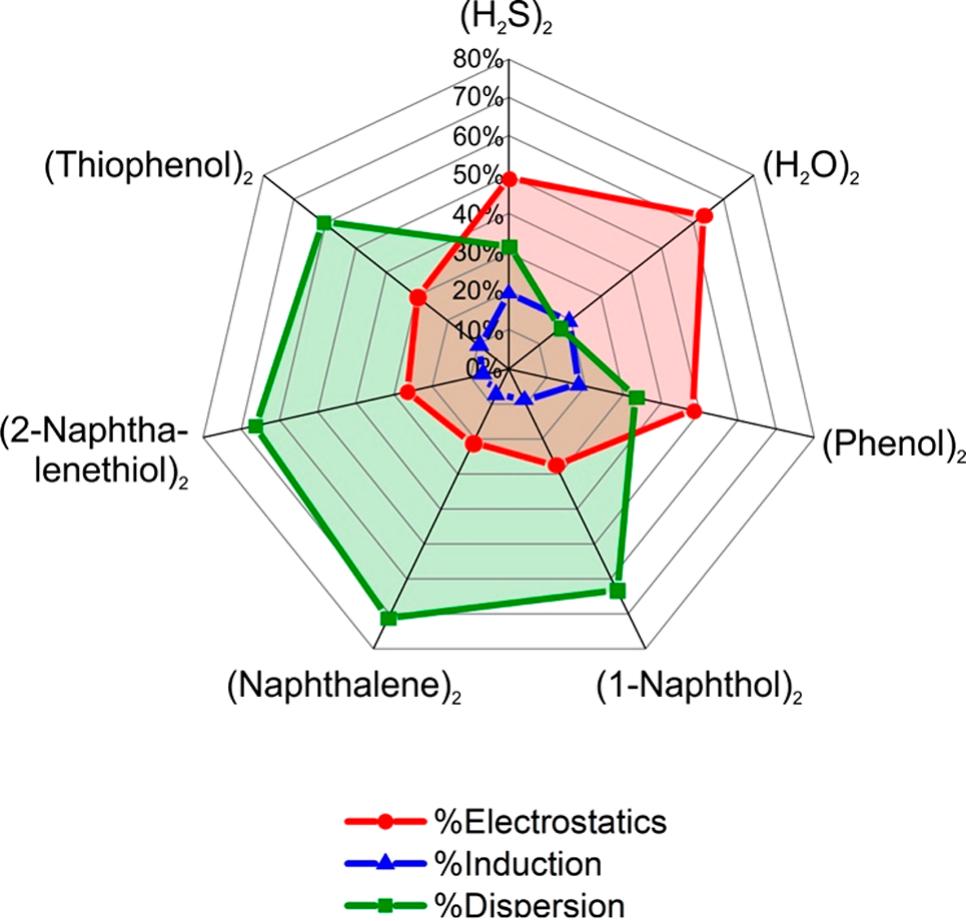
SAPT energy decomposition for the 2-naphthalenethiol dimer and related complexes. The radar chart shows the percentage of electrostatic, induction, and dispersion energy compared to the total stabilization energy in each complex. See Table 2 and Figure S12 for the total energy values.

In conclusion, gas-phase intermolecular clusters constitute chemically specific models of molecular aggregation. However, most studies have focused on hydrogen-bonding, and the observation of π-stacking aggregates has remained elusive. The detection of the 2-naphthalenethiol dimer significantly expands our understanding of π-stacking on substituted bicyclic aromatic hydrocarbons, offering valuable insight about their electronic properties, energetics, noncovalent interactions, and internal dynamics. Several conclusions are worth noticing. The 2-naphthalenethiol dimer maintains the π-stacking arrangement predicted for naphthalene,^36^ but its shallow PES increases in complexity and difficulty, offering a large number of low-lying isomers in the sub-kJ mol^–1^ window, difficult to model computationally. The experimental detection of two isomers of the dimer constitutes the first rotational observation of π-stacking isomerism and evidences the important internal dynamics observable in the gas phase, previously noticed in the dimers of benzene,^27,28^ difluorobenzene,^25^ and thiophenol.^30^ The observed dimers of 2-naphthalenethiol have structural resemblances with those of naphthalene and naphthol, one of them sharing the *C*_2_-symmetric arrangement of the naphthalene dimer global minimum. However, the substituent effect of the thiol group is mostly noticed in the increased dimer stabilization compared to the naphthalene or naphthol dimers (increase of +53% or +12%, respectively, in complexation energy). The energy decomposition analysis confirms the dominant character of dispersion forces, common to the three π-stacking structures and distinctive element with respect to hydrogen-bonded clusters. π-Stacking interactions are described through NCI plots as extended diffuse interactions, with localized attractive pockets compatible with the recent descriptions balancing dispersion forces and Pauli repulsion.^12^ Finally, the introduction of polar groups with associated bond dipoles proves an interesting chemical tool to gauge the molecular balance between the increased electrostatic forces, dispersion, and Pauli repulsion, thus offering different scenarios to test π-stacking forces. Considering that both repulsion and dispersion have size-dependent magnitudes, new experiments could now be devised examining the impact of substituents and heteroatoms in other polycyclic aromatic hydrocarbons. In this effort, the collaboration with adequate dispersion-corrected quantum mechanical models will be critical, emphasizing the synergistic role of gas-phase high-resolution rotational experiments.

## Supporting Information Available

Experimental and computational methods, analysis of the rotational spectrum, supplementary Figures (stable structures, microwave spectrum, rotatable drawings, interconversion barriers), and supplementary Tables (calculated energetic and rotational parameters, list of the observed rotational transitions, theoretical Cartesian coordinates) (PDF)
