# Supplementary material for: π-Stacking Isomerism in Polycyclic Aromatic Hydrocarbons: The 2-Naphthalenethiol Dimer
Source: J Phys Chem Lett. 2022 Dec 30;14(1):207–13. doi: 10.1021/acs.jpclett.2c03299 (PMC9841560; doi:10.1021/acs.jpclett.2c03299)
Supplement: Supplementary file 1 — jz2c03299_si_001.pdf [file jz2c03299_si_001.pdf]

π-Stacking Isomerism in Polycyclic Aromatic Hydrocarbons: The 2-Naphthalenethiol Dimer

SUPPORTING INFORMATION

Rizalina Tama Saragi,[1,2] Camilla Calabrese,[1] Marcos Juanes,[1,2] Ruth Pinacho,[3] José Emiliano Rubio,[3] Cristóbal Pérez,*[1] Alberto Lesarri*[1]

1 Departamento de Química Física y Química Inorgánica, Facultad de Ciencias, Universidad de Valladolid
Paseo de Belén, 7, 47011 Valladolid (Spain)
E-mail: cristobal.perez@uva.es, alberto.lesarri@uva.es

2 Present address: Institut für Ionenphysik und Angewandte Physik, Universität Innsbruck
Technikerstr. 25/4. OG, 6020 Innsbruck (Austria)

3 Departamento de Electrónica, ETSIT, Universidad de Valladolid
Paseo de Belén, 11, 47011 Valladolid (Spain)

TABLE OF CONTENTS

1. **Experimental and Computational Methods**
2. **Rotational Spectrum**
3. **Supplementary Figures**

Figure S1. Predicted Gibbs energies (B3LYP-D3(BJ), kJ mol^{-1}) and interconversion barrier between the two conformers (*cis* and *trans*) of 2-naphthalenethiol.

Figure S2. The nineteen most stable structures of the 2-naphthalenethiol dimer using B3LYP-D3(BJ)/def2-TZVP. The relative energy differences are indicated in blue (ZPE-corrected, kJ mol^{-1}). The notation reflects the conformation of the monomer (C for *cis*, T for *trans*). Dimers 1 and 5 have C_2-symmetry, the rest of the dimers have C_1-symmetry.

Figure S3. Conformational stability of the first seven isomers of the 2-naphthalenethiol dimer, using B3LYP-D3(BJ)/def2-TZVP (light blue trace, Table S1), B3LYP-D3(BJ)/def2-TZVPD (red trace, Table S2), B3LYP-D3(BJ)/cc-pVTZ (yellow trace, Table S3), ωB97XD/def2-TZVP (grey trace, Table S4), RI-SCS-MP2/def2-TZVP (green trace, Table S6) and DLPNO-CCSD(T)/def2-TZVP (dark blue, Table S7). The DFT methods (except ωB97XD) predict isomer CC-1 as global minimum, while MP2 and DLPNO-CCSD(T) favor isomer CC-2 as most stable.

Figure S4. The microwave spectrum of 2-naphthalenethiol and an expanded section illustrating rotational transitions from the two isomers of the dimer (CC-1 and CC-2).

Figure S5. Rotatable 3D figure of the CC-1 isomer of the 2-naphthalenethiol dimer (B3LYP-D3(BJ)/def2-TZVP).

Figure S6. Rotatable 3D figure of the CC-2 isomer of the 2-naphthalenethiol dimer (B3LYP-D3(BJ)/def2-TZVP).

Figure S7. Inter-ring torsion angles in isomer CC-1 of the 2-naphthalenethiol dimer and comparison with the most stable isomer of the naphthalene dimer and isomer a of the naphthol dimer, using B3LYP-D3(BJ)/def2-TZVP calculations.

Figure S8. Inter-ring torsion angles in isomer CC-2 of the 2-naphthalenethiol dimer and comparison with the observed "V-shape" dimer of 1-naphthol and the D_{2d} isomer of the naphthalene dimer, using B3LYP-D3(BJ)/def2-TZVP calculations.

Figure S9. Distances between the molecular planes of 2-naphthalenethiol for the most stable dimer structures, according to B2PLYP-D3(BJ)/def2-TZVP calculations in Table S4. The closest distances between the two planes are indicated by green dashed lines. The S-H···S hydrogen bond is present only in dimers 6, 7, and 9 (purple dashed lines).

Figure S10. Interconversion barrier between isomers CC-1 and CC-2. The intermediates and transition states for the interconversion barrier between the two most stable dimers were located using the Global Reaction Route Mapping (GRRM) program, linked to Gaussian16. Transition state structures were optimized as saddle points at the B3LYP-D3(BJ)/def2-TZVP level of calculation. Intrinsic reaction coordinate (IRC) calculations were performed at the same level.

Figure S11. Comparison of the Johnson-Contreras reduced electronic density gradient s $\left(= \frac{1}{1(3\pi^2)^{1/3}} \frac{|\nabla\rho|}{\rho^{4/3}}\right)$ versus the signed electronic density (= sign (λ_2) ρ) for the dimer of 2-naphthalenethiol (upper panel), and the dimers of phenol and thiophenol (lower panels). Plot minima at negative horizontal coordinates suggest attractive forces. Repulsive interactions (positive horizontal coordinates) are associated to destabilizing forces and ring critical points. The characteristic negative minima associated to the O-H···O and S-H···S hydrogen bonds in the phenol and thiophenol dimers are absent for the naphthalenethiol dimer.

Figure S12. Comparison of the SAPT energy decomposition of Table S13 for the 2-naphthalenethiol dimer and related complexes, showing the total stabilization energy (orange) and the attractive contributions due to electrostatic (red), induction (blue) and dispersion forces (green). The larger dispersion component is evident for the homodimers formed by π-stacking interactions, while the hydrogen bonds are associated to dominant electrostatic contributions. The larger complexation energy of the dimer of 2-naphthalenethiol vs naphthalene is also noticeable.

4. Supplementary Tables

Table S1. Conformational search and prediction of the rotational and energetic parameters for the 2-naphthalenethiol dimer using the B3LYP-D3(BJ) method and a polarized triple-ζ basis set (def2-TZVP).

Table S2. Conformational search and prediction of the rotational and energetic parameters for the 2-naphthalenethiol dimer using the B3LYP-D3(BJ) method and a triple-ζ basis set including polarized and diffuse functions (def2-TZVPD).

Table S3. Reoptimization of the ten most stable isomers of the 2-naphthalenethiol dimer using the ωB97XD method and the def2-TZVP basis set.

Table S4. Reoptimization of the six most stable isomers of the 2-naphthalenethiol dimer using the B2PLYP-D3(BJ) method and the def2-TZVP basis set.

Table S5. Reoptimization of the six most stable isomers of the 2-naphthalenethiol dimer using the MP2 method and the def2-TZVP basis set.

Table S6. Single-point energy calculation using DLPNO-CCSD(T)/def2-TZVP for the B3LYP-D3(BJ) geometries of Table S1.

Table S7. Observed rotational transitions of isomer 1 (CC-1) of the 2-naphtalenethiol dimer (Freq.), together with the differences with the calculated transitions (o.-c.) for the fit of Table 1 and estimated frequency uncertainties (unc.). All values in MHz.

Table S8. Observed rotational transitions of isomer 2 (CC2) of the 2-naphtalenethiol dimer (Freq.), together with the differences with the calculated transitions (o.-c.) for the fit of Table 1 and estimated frequency uncertainties (unc.). All values in MHz.

Table S9. Equilibrium coordinates for isomer CC-1 of the 2-naphthalenethiol dimer, according to the prediction with B2PLYP-D3(BJ) /def2TZVP.

Table S10. Equilibrium coordinates for isomer CC-2 of the 2-naphthalenethiol dimer, according to the prediction with B2PLYP-D3(BJ) /def2TZVP.

Table S11. Rotational constants of the observed dimers of 2-naphtalenethiol compared to four theoretical predictions with B3LYP-D3(BJ), ωB97XD, B2PLYPD3(BJ) and MP2 using def2-TZVP as basis set, and their relative percentage differences.

Table S12. Rotational constants of the observed dimers of 2-naphtalenethiol compared to the theoretical model B3LYP-D3(BJ) using either the def2-TZVP or def2-TZVPD basis sets, and their relative percentage differences.

1. Experimental and Computational Methods

The investigation of the 2-naphthalenethiol dimer used a commercial sample (>98% GC) which required no further purification. The solid sample (m.p. 80-81°C, b.p. 92-94°C) was vaporized *in situ* at temperatures of ca. 110°C using a heatable solenoid injector (Parker, Series 9) with a pinhole (ϕ=0.8 mm) conical nozzle. Pressurization with neon at backing pressures of 1.0-3.5 bar, followed by pulsed expansion into a high-vacuum chamber (10^{-6}-10^{-7} mbar), produced the supersonic jet. The typical gas pulses had a duration of 900 μs. Intermolecular three-body (or larger) collisions at the nozzle create the dimer species, which are later stabilized and probed in the (rovibrationally cooled) silence zone of the jet. The dimer was detected with a broadband chirped-pulsed Fourier transform microwave (CP-FTMW) spectrometer[1–3] working in the 2-8 GHz frequency region. The spectrometer uses a direct-digital design,[4] based on short chirped-pulse excitation produced with a fast arbitrary waveform generator (25 GSamples/s). For a typical operation a sequence of five (4 μs) chirp pulses, amplified to 20 W, excited the full 6 GHz bandwidth. The MW radiation induces a macroscopic polarization by a fast-passage mechanism,[5] followed by rotational decoherence and emission of a free-induction decay. The molecular signal (40 μs/excitation pulse) is amplified and recorded in the time domain with a digital oscilloscope (25 GSamples/s). The frequency-domain spectrum was obtained with a Fourier transformation and a Kaiser-Bessel apodization window. The resulting linewidths (full-width at half-maximum) are of ca. 100 kHz. For the present experiment 1 M averages were recorded (repetition rate of 5 Hz). The uncertainty of the frequency measurements is estimated to be better than 20 kHz.

The experiment was complemented with electronic structure calculations, mostly based on density functional theory (DFT). The initial dimer geometries were obtained with an hybrid Monte Carlo/low-mode[6] conformational search based on molecular mechanics (MMFFs[7]). Additional structures were then added manually based on chemical arguments. All isomers were later fully optimized with the B3LYP[8] and ωB97XD[9] hybrid methods and a polarized triple-ζ def2-TZVP basis set.[10] D3 dispersion corrections[11] and a Becke-Johnson damping function[11] where added to B3LYP, while ωB97XD includes a variant of the D2 method. The quality of the basis set was checked by new B3LYP-D3(BJ) optimizations adding diffuse functions (def2-TZVPD) or correlation consistent (cc-pVTZ) basis sets. In order to check the computational consistency, the seven most stable isomers were reoptimized with two more

methods, including double-hybrid B2PLYP[12] DFT and spin-component-scaled second-order Moller-Plesset perturbation theory SCS-MP2[13,14] within the resolution-of-identity (RIJCOSX) approximation.[15]

Finally, the isomer energies were recalculated with domain-based local pair-natural orbital coupled cluster perturbative triple-excitations method (DLPNO-CCSD(T)) calculations with the def2-TZVPP basis set and the resolution-of-identity (RIJCOSX) approximation.[15]

Harmonic vibrational frequency calculations confirmed that all stationary points were minima of the PES. However, the molecular size of this system prevented frequency calculations at the B2PLYP, RI-SCS-MP2 and DLPNO-CCSD(T) with our present computational resources. Complexation energies included the basis set superposition error (BSSE), corrected with the counterpoise method.[16] The quantum mechanical calculations were implemented in Gaussian16,[17] except for the SCS-MP2 and DLPNO-CCSD(T), which used ORCA.[18] The intermediates and transition states for the interconversion barriers between the most stable dimers were located by means of the Global Reaction Route Mapping (GRRM) program,[19] linked to Gaussian16. Transition state structures were optimized as saddle points at the B3LYP-D3(BJ)/def2-TZVP level of calculation. Intrinsic reaction coordinate (IRC) calculations were performed at the same level. Noncovalent interactions were explored using energy decomposition analysis and symmetry-adapted perturbation theory (SAPT[20]), implemented in PSI4.[21] The SAPT calculation used a 2+(3) approximation and the aug/cc-PVDZ basis set. Additionally, the topology of the electron density was analysed with the Johnson-Contreras NCIplot method.[22,23]

2. Analysis of the Rotational Spectrum

The microwave spectrum in Figure S3 was dominated by the monomer and its neon cluster, not reported here. Both *cis*- and *trans*-2-naphthalenethiol were identified, including the parent and all ^{13}C and ^{34}S monosubstituted isotopologues. In addition, two weaker spectral signatures were detected, confirming the competition between two isomers of the dimer. Both spectra correspond to near-prolate asymmetric rotors, but they exhibit marked differences. The first spectrum consisted of only R-branch (J+1←J) transitions activated by the μ_c electric dipole moment. This feature is characteristically associated to the presence of a *c*-symmetry axis, cancelling other dipole components. Conversely, μ_b- and μ_c-type R-branch transitions were detected for the second species,

excluding the presence of symmetry elements. This spectrum displayed a coalescence of the *b-c* asymmetry quartets associated to the large-mass high-*J* asymptotic limit,[24] which prevented a calculation of relative intensities between both isomers. No tunnelling hyperfine effects were observed, and the spectra were reproduced to experimental uncertainty using a semirigid model implemented in the Watson (S-reduced, I^r reduction) rotational Hamiltonian.[25] The experimental dataset, comprising a total of more than 250 transitions, is presented in Tables S8-S9. The derived experimental parameters are collected in Table 1. No other species of reasonable intensity were identified. Plausible ^{13}C or ^{34}S isotopologues of the dimer were undetectable in natural abundance. Unassigned lines remained in the spectrum, which be originated by minor isotopologues, other clusters or impurities.

The rotational constants of the two isomers are very similar, making the conformational assignment apparently difficult. However, the consideration of the values of (*B*-*C*) and (*B*+*C*) rotational constants, the electric dipole moments and the centrifugal distortion constants produced an unequivocal identification. In particular, the presence of a symmetry axis matches the predictions for the global minimum CC-1 (in no other case $\mu_c >> \mu_a > \mu_b$). Similarly, the observed transitions of the second isomer match the predictions for CC-2. In this case the μ_a (=0.2 D) component is not zero, but its small magnitude prevented the observation of these transitions. Rotatable 3D figures and coordinates for both isomers are shown in Figures S5-S6 and Tables S10-S11. The comparison between experiment and theory in Table 1 gives a good structural agreement for the B3LYP-D3(BJ) model, with relative differences in the rotational constants of 0.7-3.1% for isomer CC-1 and 1.5-1.9% for isomer CC-2. A comparison with the alternative theoretical models is shown in Table S12. Interestingly, neither ωB97XD nor the double-hybrid B2PLYP offered significative improvements over B3PLYP in structural terms. Expansion of the basis set to diffuse functions def2-TZVPD or cc-pVTZ was not beneficial either (Table S13). The behavior of SCS-MP2 was comparable to DFT, while over representation of dispersion in normal MP2 (not reported),[14,26] resulted in larger uncertainties unsuitable for the spectroscopic work (i.e., 3.9-10.0% for CC-2).

References

(1) Shipman, S. T.; Pate, B. H. New Techniques in Microwave Spectroscopy. In *Handbook of High-resolution Spectroscopy*; Merkt, F., Quack, M., Eds.; Major Reference Works; John Wiley & Sons, Ltd: New York, 2011; pp 801–828. https://doi.org/10.1002/9780470749593.hrs036.

(2) Grabow, J.-U. Fourier Transform Microwave Spectroscopy Measurement and Instrumentation. In *Handbook of High-resolution Spectroscopy*; Merkt, F., Quack, M., Eds.; John Wiley & Sons, Ltd: New York, 2011; pp 723–799. https://doi.org/10.1002/9780470749593.hrs037.

(3) Caminati, W.; Grabow, J.-U. Advancements in Microwave Spectroscopy. In *Frontiers and Advances in Molecular Spectroscopy*; Laane, J., Ed.; Elsevier Inc., 2018; pp 569–598. https://doi.org/10.1016/B978-0-12-811220-5.00018-6.

(4) Neill, J. L.; Shipman, S. T.; Alvarez-Valtierra, L.; Lesarri, A.; Kisiel, Z.; Pate, B. H. Rotational Spectroscopy of Iodobenzene and Iodobenzene-Neon with a Direct Digital 2-8 GHz Chirped-Pulse Fourier Transform Microwave Spectrometer. *J. Mol. Spectrosc.* **2011**, *269* (1), 21–29. https://doi.org/10.1016/j.jms.2011.04.016.

(5) McGurk, J. C.; Schmalz, T. G.; Flygare, W. H. Fast Passage in Rotational Spectroscopy: Theory and Experiment. *J. Chem. Phys.* **1974**, *60* (11), 4181–4188. https://doi.org/10.1063/1.1680886.

(6) Keserû, G. M.; Kolossváry, I. Fully Flexible Low-Mode Docking: Application to Induced Fit in HIV Integrase. *J. Am. Chem. Soc.* **2001**, *123* (50), 12708–12709. https://doi.org/10.1021/ja0160086.

(7) Halgren, T. A. MMFF VI. MMFF94s Option for Energy Minimization Studies. *J. Comput. Chem.* **1999**, *20* (7), 720–729. https://doi.org/10.1002/(SICI)1096-987X(199905)20:7<720::AID-JCC7>3.0.CO;2-X.

(8) Becke, A. D. Density-Functional Thermochemistry. III. The Role of Exact Exchange. *J. Chem. Phys.* **1993**, *98* (7), 5648–5652. https://doi.org/10.1063/1.464913.

(9) Chai, J.-D.; Head-Gordon, M. Long-Range Corrected Hybrid Density Functionals with Damped Atom–Atom Dispersion Corrections. *Phys. Chem. Chem. Phys.* **2008**, *10* (44), 6615. https://doi.org/10.1039/b810189b.

(10) Weigend, F.; Ahlrichs, R. Balanced Basis Sets of Split Valence, Triple Zeta Valence and Quadruple Zeta Valence Quality for H to Rn: Design and Assessment of Accuracy. *Phys. Chem. Chem. Phys.* **2005**, *7* (18), 3297. https://doi.org/10.1039/b508541a.

(11) Grimme, S.; Ehrlich, S.; Goerigk, L. Effect of the Damping Function in Dispersion Corrected Density Functional Theory. *J. Comput. Chem.* **2011**, *32* (7), 1456–1465. https://doi.org/10.1002/jcc.21759.

(12) Grimme, S.; Neese, F. Double-Hybrid Density Functional Theory for Excited Electronic States of Molecules. *J. Chem. Phys.* **2007**, *127* (15), 1–18. https://doi.org/10.1063/1.2772854.

(13) Møller, C.; Plesset, M. S. Note on an Approximation Treatment for Many-Electron Systems. *Phys. Rev.* **1934**, *46* (7), 618–622. https://doi.org/10.1103/PhysRev.46.618.

(14) Riley, K. E.; Platts, J. A.; Řezáč, J.; Hobza, P.; Hill, J. G. Assessment of the Performance of MP2 and MP2 Variants for the Treatment of Noncovalent Interactions. *J. Phys. Chem. A* **2012**, *116* (16), 4159–4169. https://doi.org/10.1021/jp211997b.

(15) Riplinger, C.; Sandhoefer, B.; Hansen, A.; Neese, F. Natural Triple Excitations in Local Coupled Cluster Calculations with Pair Natural Orbitals. *J. Chem. Phys.* **2013**, *139* (13), 1–13. https://doi.org/10.1063/1.4821834.

(16) Boys, S. F.; Bernardi, F. The Calculation of Small Molecular Interactions by the Differences of Separate Total Energies. Some Procedures with Reduced Errors. *Mol. Phys.* **1970**, *19* (4), 553–566. https://doi.org/10.1080/00268977000101561.

(17) Frisch, M. J.; Trucks, G. W.; Schlegel, H. B.; Scuseria, G. E.; Robb, M. A.; Cheeseman, J. R.; Scalmani, G.; Barone, V.; Petersson, G. A.; Nakatsuji, H.; Li, X.; Caricato, M.; Marenich, A. V.; Bloino, J.; Janesko, B. G.; Gomperts, R.; Mennucci, B.; Hratchian, H. P.; Ortiz, J. V.; Izmaylov, A. F.; Sonnenberg, J. L.; Williams; Ding, F.; Lipparini, F.; Egidi, F.; Goings, J.; Peng, B.; Petrone, A.; Henderson, T.; Ranasinghe, D.; Zakrzewski, V. G.; Gao, J.; Rega, N.; Zheng, G.; Liang, W.; Hada, M.; Ehara, M.; Toyota, K.; Fukuda, R.; Hasegawa, J.; Ishida, M.; Nakajima, T.; Honda, Y.; Kitao, O.; Nakai, H.; Vreven, T.; Throssell, K.; Montgomery Jr., J. A.; Peralta, J. E.; Ogliaro, F.; Bearpark, M. J.; Heyd, J. J.; Brothers, E. N.; Kudin, K. N.; Staroverov, V. N.; Keith, T. A.; Kobayashi, R.; Normand, J.; Raghavachari, K.; Rendell, A. P.; Burant, J. C.; Iyengar, S. S.; Tomasi, J.; Cossi, M.; Millam, J. M.; Klene, M.; Adamo, C.; Cammi, R.; Ochterski, J. W.; Martin, R. L.; Morokuma, K.; Farkas, O.; Foresman, J. B.; Fox, D. J. Gaussian 16, Rev. C.01. Gaussian, Inc: Wallingford CT 2016.

(18) Neese, F. Software Update: The ORCA Program System, Version 4.0. *WIREs Comput. Mol. Sci.* **2018**, *8* (1), 1. https://doi.org/10.1002/wcms.1327.

(19) Ohno, K.; Maeda, S. A Scaled Hypersphere Search Method for the Topography of Reaction Pathways on the Potential Energy Surface. *Chem. Phys. Lett.* **2004**, *384* (4–6), 277–282. https://doi.org/10.1016/j.cplett.2003.12.030.

(20) Jeziorski, B.; Moszynski, R.; Szalewicz, K. Perturbation Theory Approach to Intermolecular Potential Energy Surfaces of van Der Waals Complexes. *Chem. Rev.* **1994**, *94* (7), 1887–1930. https://doi.org/10.1021/cr00031a008.
(21) Parrish, R. M.; Burns, L. A.; Smith, D. G. A.; Simmonett, A. C.; DePrince, A. E.; Hohenstein, E. G.; Bozkaya, U.; Sokolov, A. Y.; Di Remigio, R.; Richard, R. M.; Gonthier, J. F.; James, A. M.; McAlexander, H. R.; Kumar, A.; Saitow, M.; Wang, X.; Pritchard, B. P.; Verma, P.; Schaefer, H. F.; Patkowski, K.; King, R. A.; Valeev, E. F.; Evangelista, F. A.; Turney, J. M.; Crawford, T. D.; Sherrill, C. D. Psi4 1.1: An Open-Source Electronic Structure Program Emphasizing Automation, Advanced Libraries, and Interoperability. *J. Chem. Theory Comput.* **2017**, *13* (7), 3185–3197. https://doi.org/10.1021/acs.jctc.7b00174.
(22) Johnson, E. R.; Keinan, S.; Mori-Sánchez, P.; Contreras-García, J.; Cohen, A. J.; Yang, W. Revealing Noncovalent Interactions. *J. Am. Chem. Soc.* **2010**, *132* (18), 6498–6506. https://doi.org/10.1021/ja100936w.
(23) Contreras-García, J.; Johnson, E. R.; Keinan, S.; Chaudret, R.; Piquemal, J. P.; Beratan, D. N.; Yang, W. NCIPLOT: A Program for Plotting Noncovalent Interaction Regions. *J. Chem. Theory Comput.* **2011**, *7* (3), 625–632. https://doi.org/10.1021/ct100641a.
(24) Watson, J. K. G. Asymptotic Energy Levels of a Rigid Asymmetric Top. *Mol. Phys.* **2007**, *105* (5–7), 679–688. https://doi.org/10.1080/00268970701241672.
(25) Watson, J. K. G. Aspects of Quartic and Sextic Centrifugal Effects on Rotational Energy Levels. In *Vibrational Spectra and Structure, vol. 6*; Durig, J. R., Ed.; Elsevier B.V.: Amsterdam, 1977; pp 1–89.
(26) Seifert, N. A.; Steber, A. L.; Neill, J. L.; Pérez, C.; Zaleski, D. P.; Pate, B. H.; Lesarri, A. The Interplay of Hydrogen Bonding and Dispersion in Phenol Dimer and Trimer: Structures from Broadband Rotational Spectroscopy. *Phys. Chem. Chem. Phys.* **2013**, *15* (27), 11468–11477. https://doi.org/10.1039/c3cp51725j.

3. Supplementary Figures

Figure S1. Predicted Gibbs energies (B3LYP-D3(BJ), kJ mol^{-1}) and interconversion barrier between the two conformers (*cis* and *trans*) of 2-naphthalenethiol.

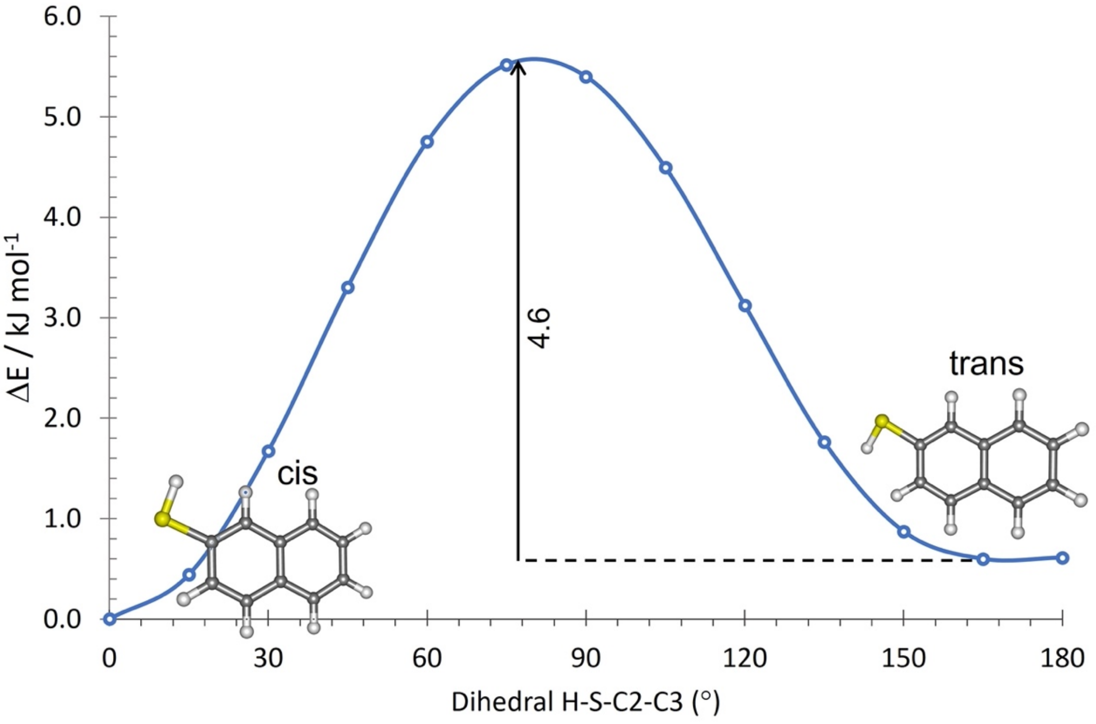

Figure S2. The nineteen most stable structures of the 2-naphthalenethiol dimer using B3LYP-D3(BJ)/def2-TZVP. The relative energy differences are indicated in blue (ZPE-corrected, kJ mol^{-1}). The notation reflects the conformation of the monomer (C for *cis*, T for *trans*). Dimers 1 and 5 have C_2-symmetry, the rest of the dimers have C_1-symmetry.

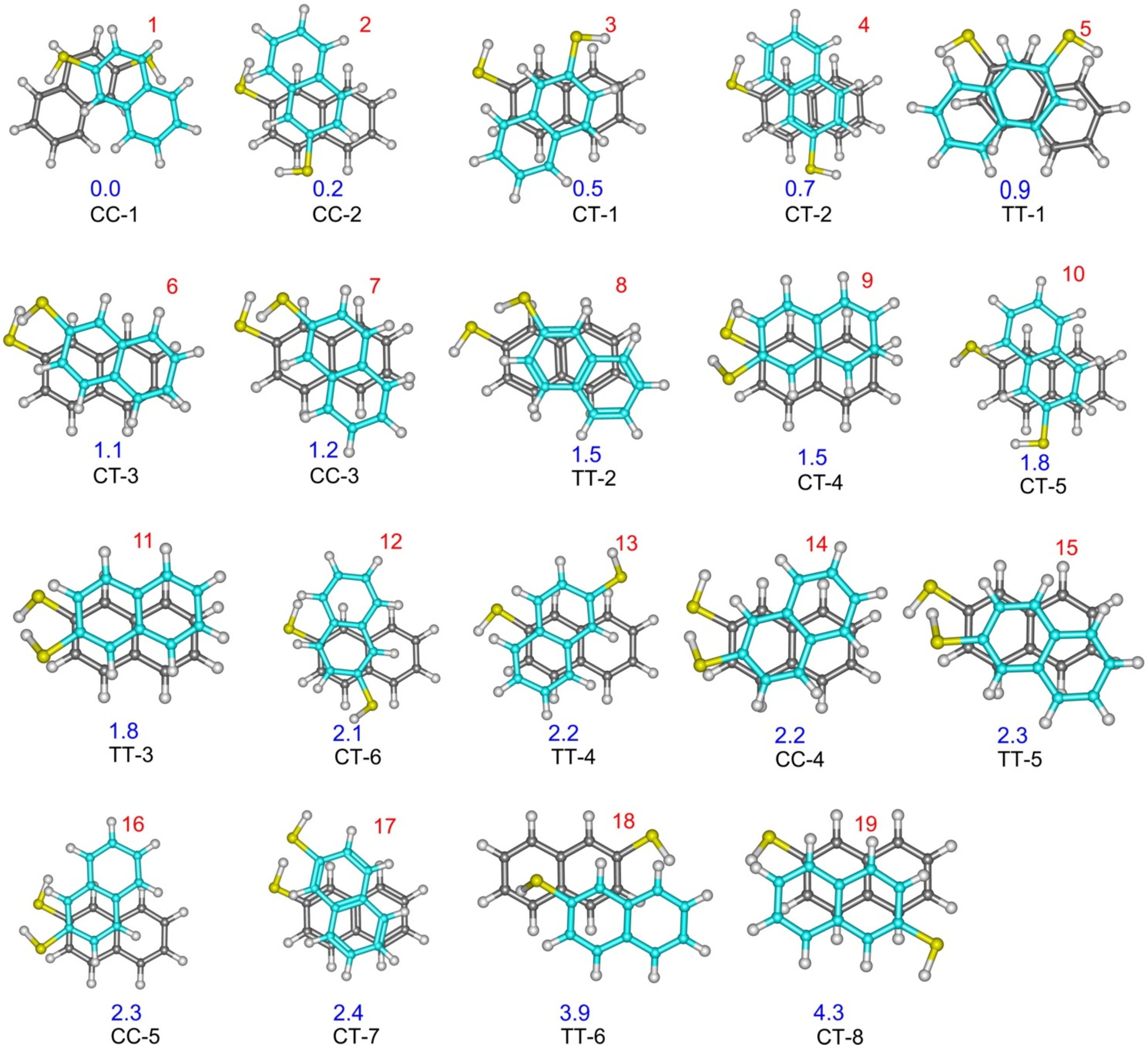

Figure S3. Conformational stability of the first seven isomers of the 2-naphthalenethiol dimer, using B3LYP-D3(BJ)/def2-TZVP (light blue trace, Table S1), B3LYP-D3(BJ)/def2-TZVPD (red trace, Table S2), B3LYP-D3(BJ)/cc-pVTZ (yellow trace, Table S3), ωB97XD/def2-TZVP (grey trace, Table S4), RI-SCS-MP2/def2-TZVP (green trace, Table S6) and DLPNO-CCSD(T)/def2-TZVP (dark blue, Table S7). The DFT methods (except ωB97XD) predict isomer CC-1 as global minimum, while MP2 and DLPNO-CCSD(T) favor isomer CC-2 as most stable.

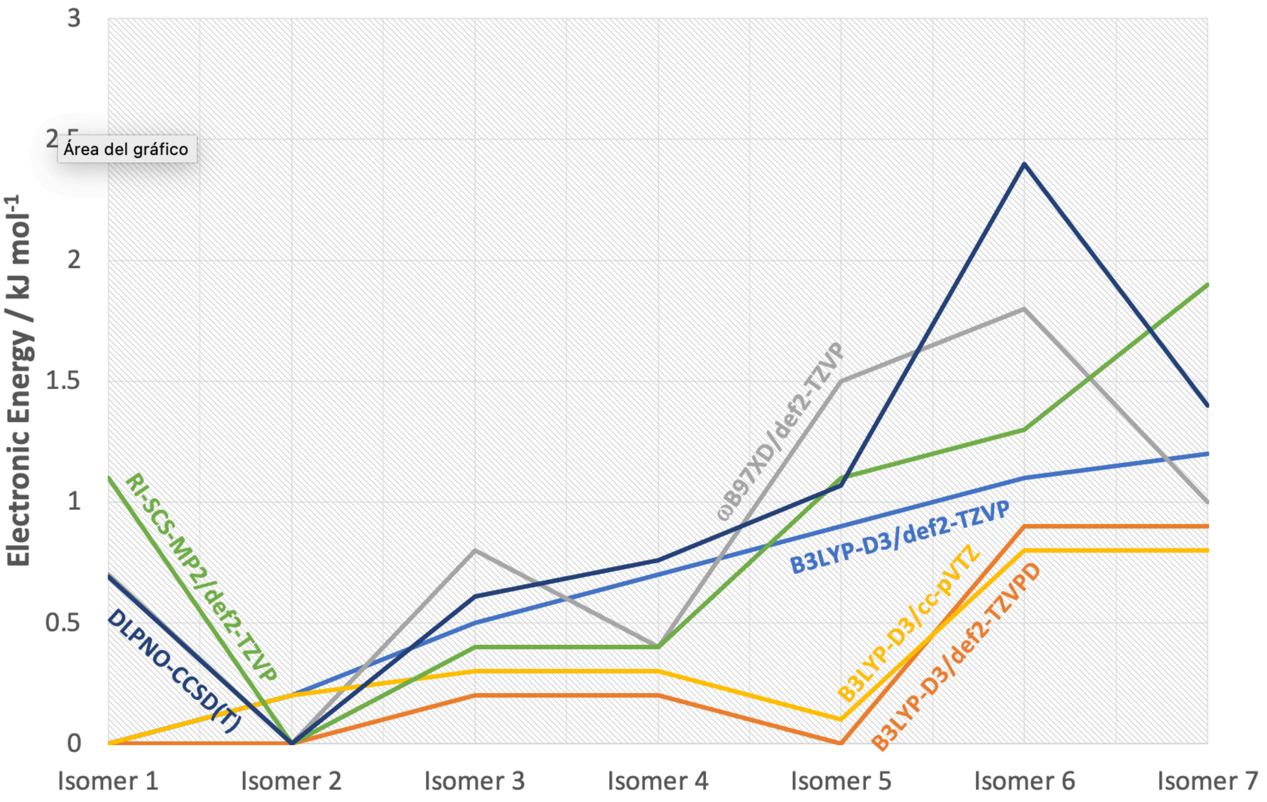

Figure S4. The microwave spectrum of 2-naphthalenethiol and an expanded section illustrating rotational transitions from the two isomers of the dimer (CC-1 and CC-2).

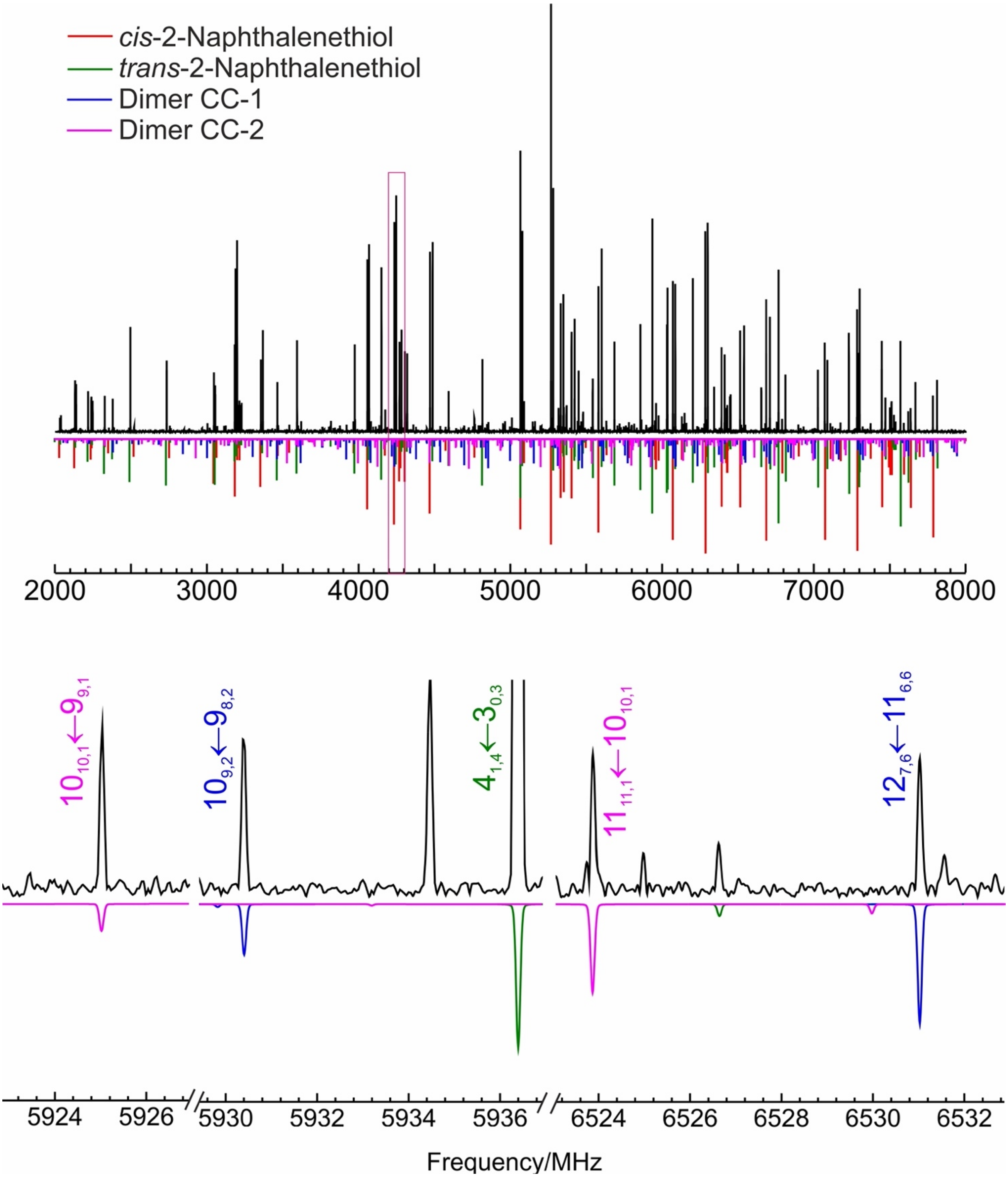

Figure S5. Rotatable 3D figure of the CC-1 isomer of the 2-naphthalenethiol dimer (B3LYP-D3(BJ)/def2-TZVP).

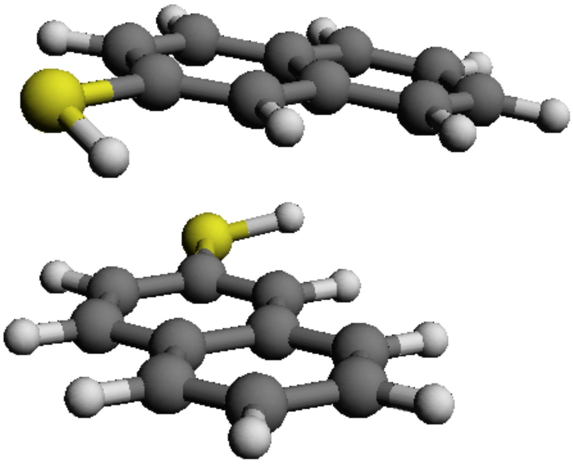

Figure S6. Rotatable 3D figure of the CC-2 isomer of the 2-naphthalenethiol dimer (B3LYP-D3(BJ)/def2-TZVP).

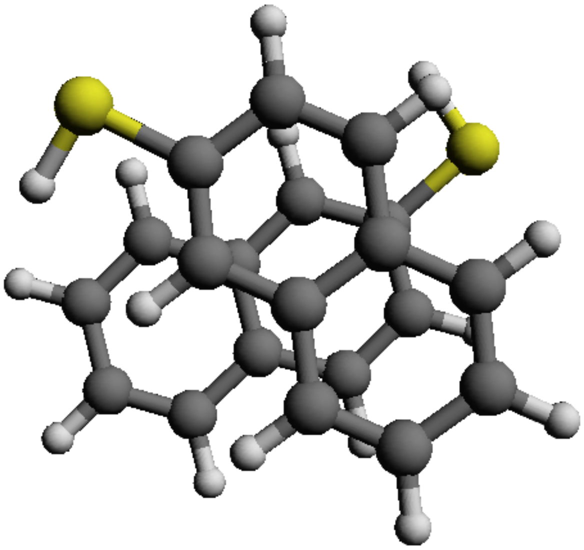

Figure S7. Inter-ring torsion angles in isomer CC-1 of the 2-naphthalenethiol dimer and comparison with the most stable isomer of the naphthalene dimer and isomer a of the naphthol dimer, using B3LYP-D3(BJ)/def2-TZVP calculations.

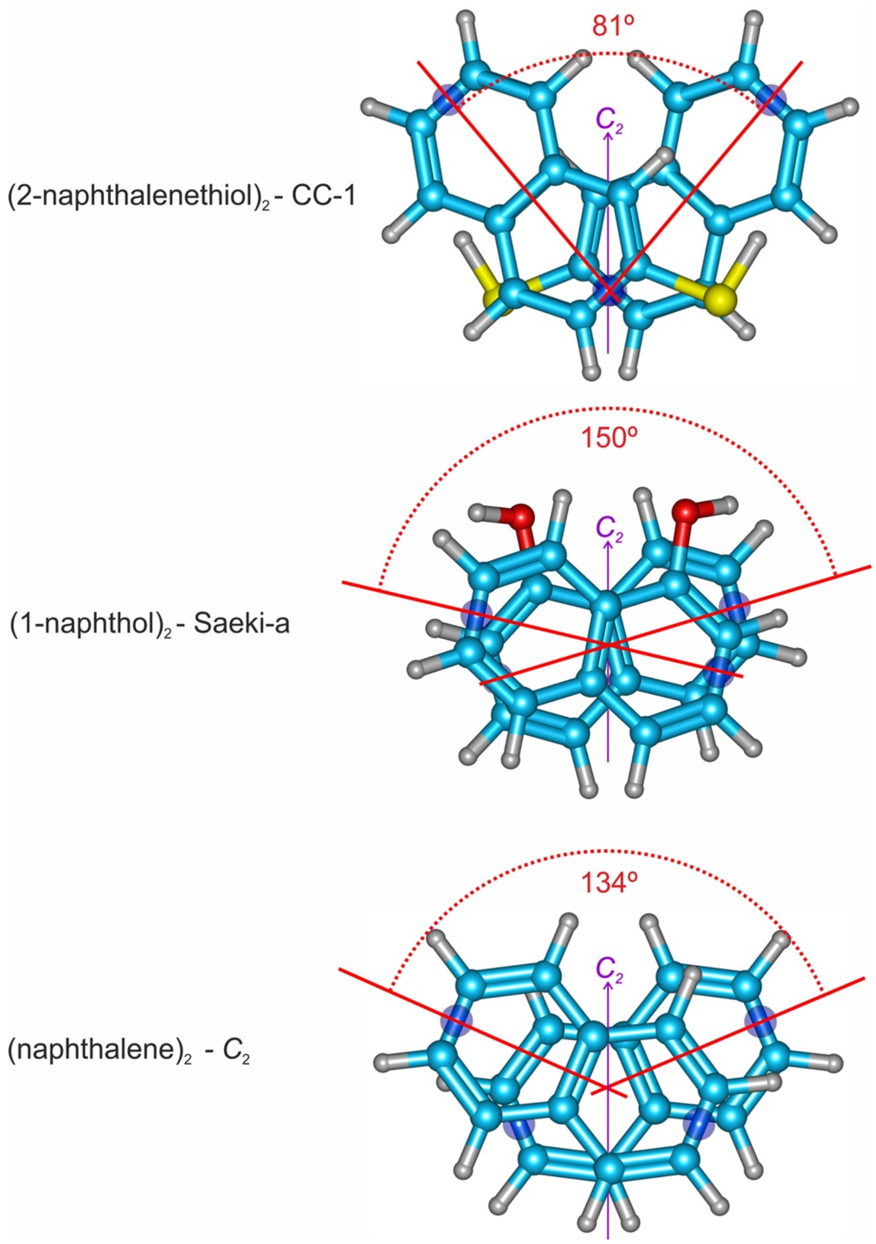

Figure S8. Inter-ring torsion angles in isomer CC-2 of the 2-naphthalenethiol dimer and comparison with the observed "V-shape" dimer of 1-naphthol and the D_{2d} isomer of the naphthalene dimer, using B3LYP-D3(BJ)/def2-TZVP calculations.

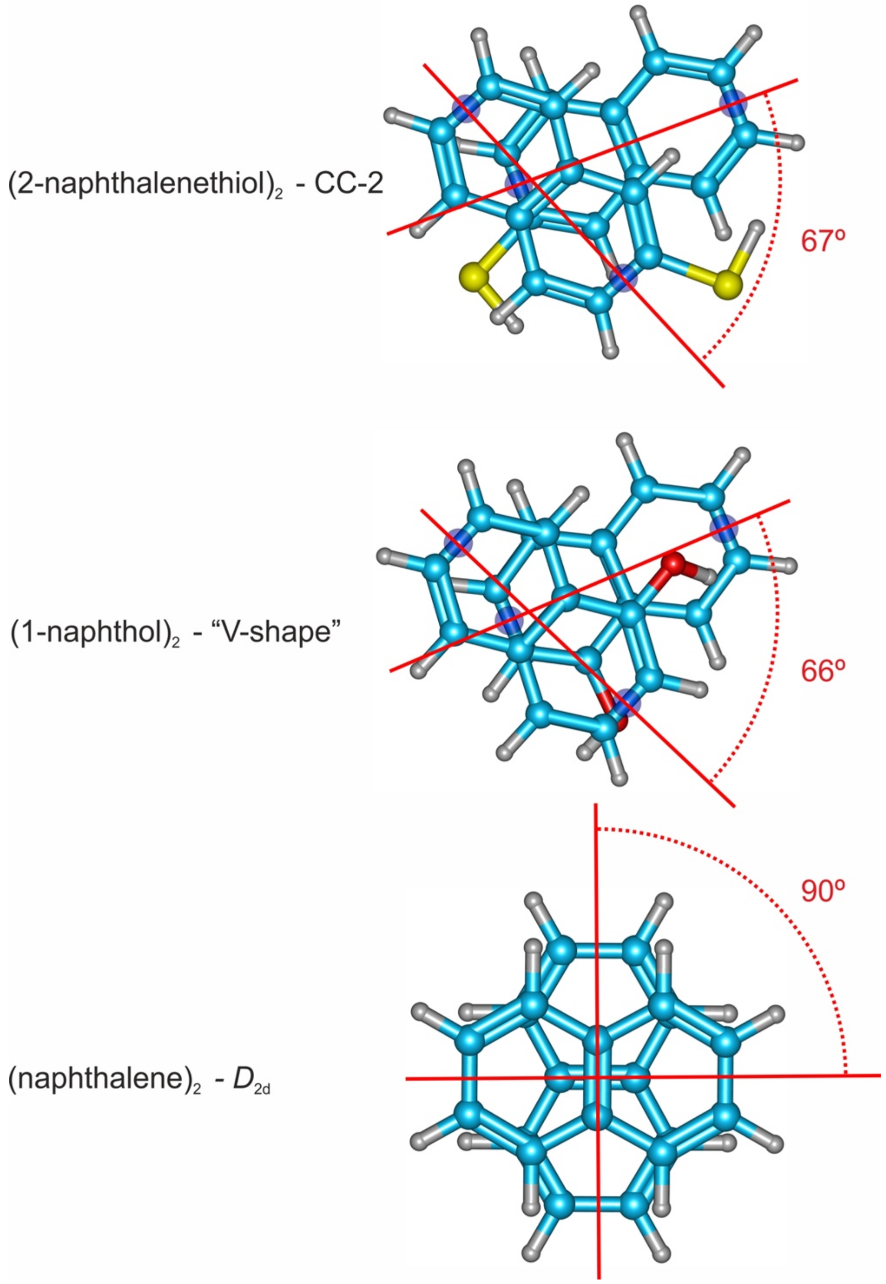

Figure S9. Distances between the molecular planes of 2-naphthalenethiol for the most stable dimer structures, according to B2PLYP-D3(BJ)/def2-TZVP calculations in Table S5. The closest distances between the two planes are indicated by green dashed lines. The S-H···S hydrogen bond is present only in dimers 6, 7, and 9 (purple dashed lines).

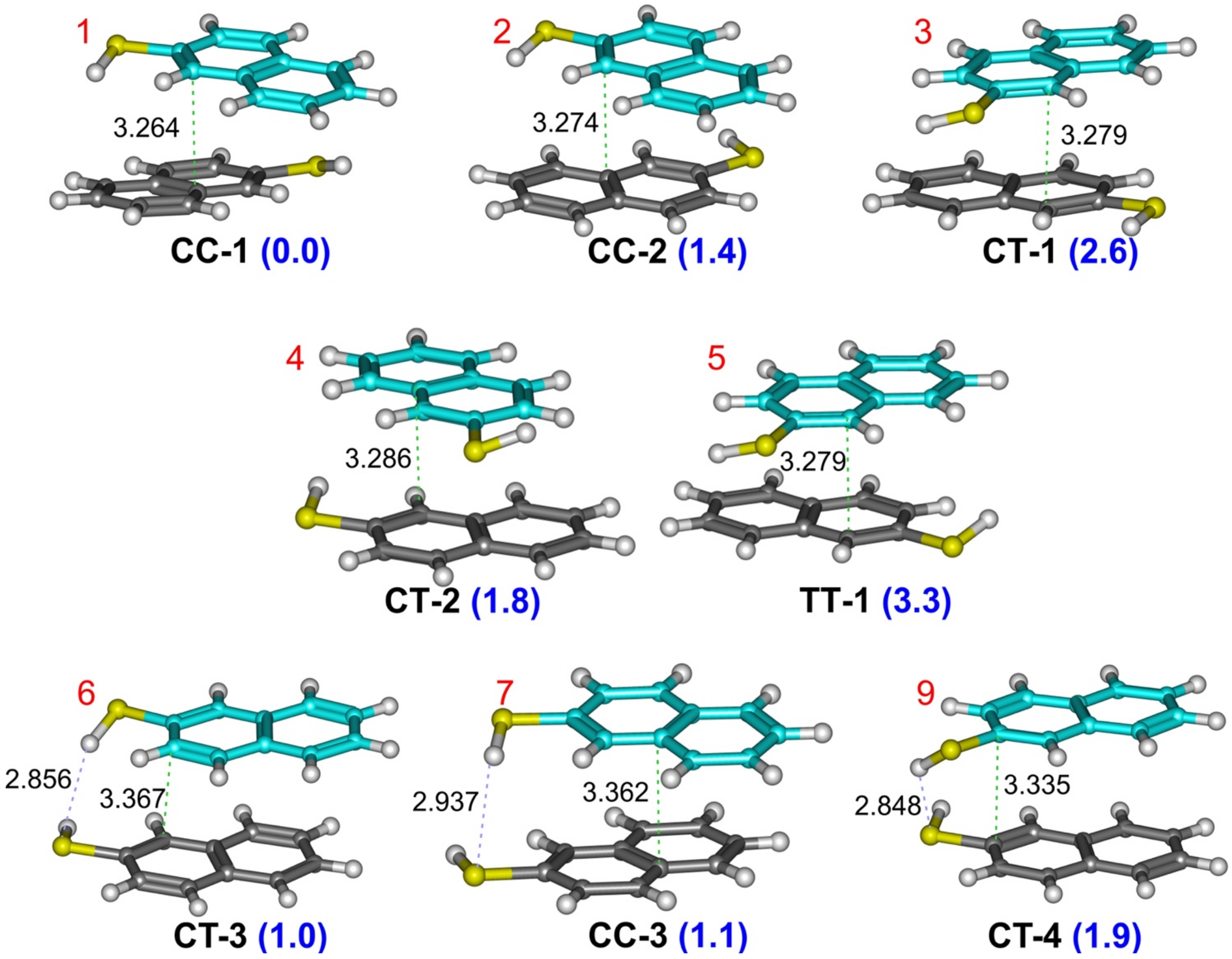

Figure S10. Interconversion barrier between isomers CC-1 and CC-2. The intermediates and transition states for the interconversion barrier between the two most stable dimers were located using the Global Reaction Route Mapping (GRRM) program, linked to Gaussian16. Transition state structures were optimized as saddle points at the B3LYP-D3(BJ)/def2-TZVP level of calculation. Intrinsic reaction coordinate (IRC) calculations were performed at the same level.

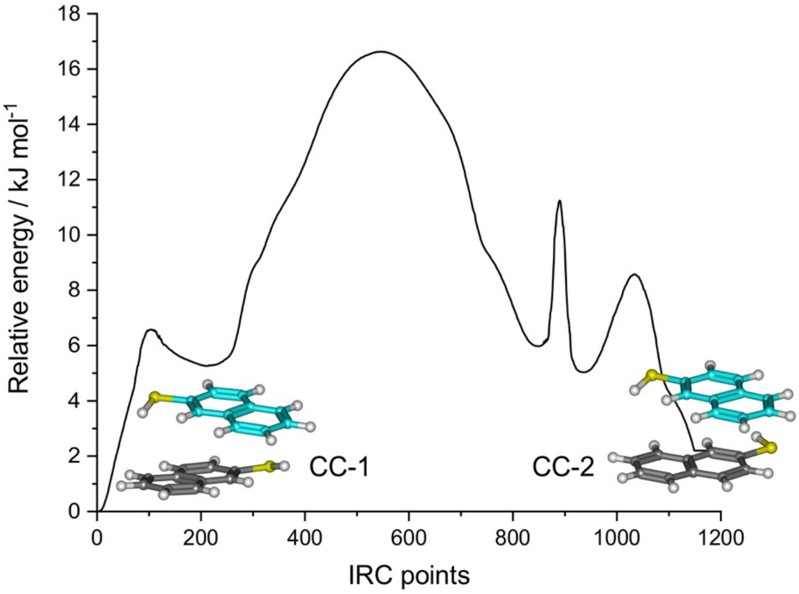

Figure S11. Comparison of the Johnson-Contreras reduced electronic density gradient s $\left(= \frac{1}{1(3\pi^2)^{1/3}} \frac{|\nabla\rho|}{\rho^{4/3}}\right)$ versus the signed electronic density (= sign (λ_2) ρ) for the dimer of 2-naphthalenethiol (upper panel), and the dimers of phenol and thiophenol (lower panels). Plot minima at negative horizontal coordinates suggest attractive forces. Repulsive interactions (positive horizontal coordinates) are associated to destabilizing forces and ring critical points. The characteristic negative minima associated to the O-H···O and S-H···S hydrogen bonds in the phenol and thiophenol dimers are absent for the naphthalenethiol dimer.

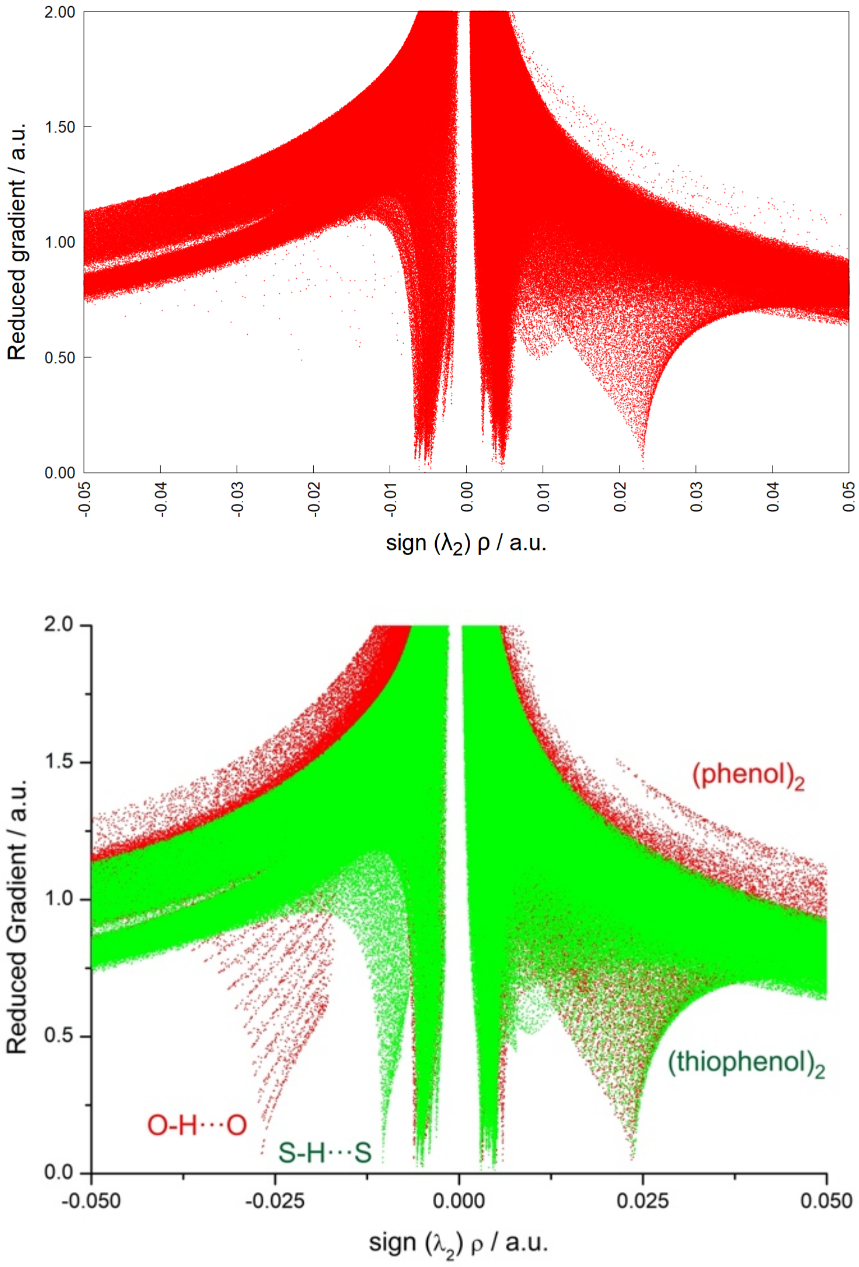

Figure S12. Comparison of the SAPT energy decomposition of Table S13 for the 2-naphthalenethiol dimer and related complexes, showing the total stabilization energy (orange) and the attractive contributions due to electrostatic (red), induction (blue) and dispersion forces (green). The larger dispersion component is evident for the homodimers formed by π-stacking interactions, while the hydrogen bonds are associated to dominant electrostatic contributions. The larger complexation energy of the dimer of 2-naphthalenethiol vs naphthalene is also noticeable.

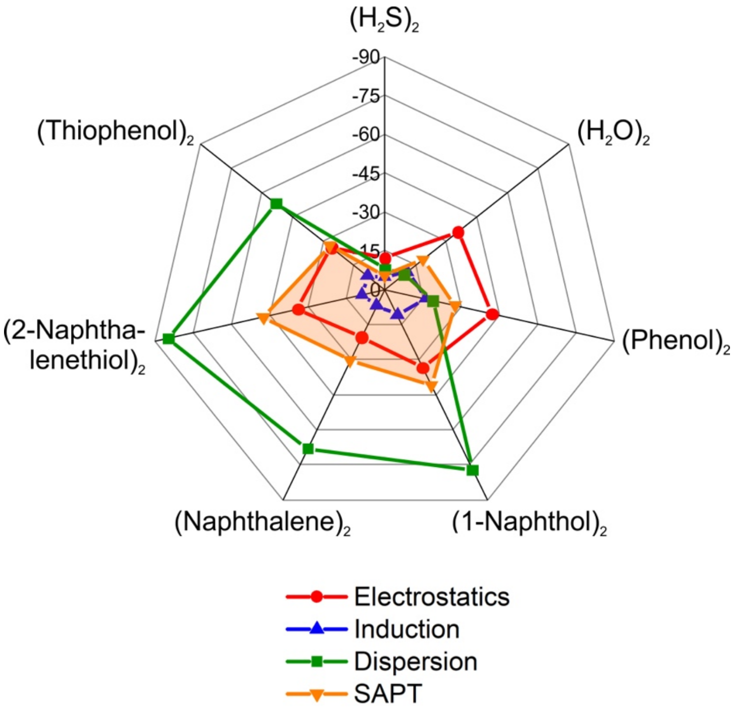

4. Supplementary Tables

Table S1. Conformational search and prediction of the rotational and energetic parameters for the 2-naphthalenethiol dimer using the B3LYP-D3(BJ) method and a polarized triple-ζ basis set (def2-TZVP).

Isomer	1 (C_2)	2	3	4	5 (C_2)	6	7
	CC-1	CC-2	CT-1	CT-2	TT-1	CT-3	CC-3
A / MHz [a]	318.1	304.2	294.6	307.2	300.3	390.1	363.6
B / MHz	233.4	250.7	245.7	249.6	242.1	228.2	231.9
C / MHz	231.3	225.7	240.9	225.6	239.4	175.3	196.9
D_J / kHz [b]	0.016	0.012	0.024	0.012	0.022	0.010	0.019
D_{JK} / kHz	-0.003	0.028	-0.037	0.032	-0.024	0.005	0.003
D_K / kHz	0.023	0.005	0.055	0.001	0.044	0.065	0.080
d_1 / kHz	-0.002	0.000	-0.007	0.000	-0.009	-0.001	0.006
d_2 / kHz	0.001	-0.001	0.002	-0.001	0.001	-0.001	-0.002
$\|\mu_a\|$ / D	0.0	0.2	1.0	0.7	0.0	1.1	1.3
$\|\mu_b\|$ / D	0.0	0.6	1.1	1.3	0.0	1.2	0.7
$\|\mu_c\|$ / D	1.3	0.6	0.6	1.2	2.1	0.0	0.3
r(S···H) / Å [c]						2.829	2.900
∠(S-H···S) / deg						140.5	132.5
centroid-centroid	3.466	3.548	3.456			3.651	3.654
ΔE_{ZPE} / kJ mol^{-1} [d]	0.0	0.2	0.5	0.7	0.9	1.1	1.2
ΔG / kJ mol^{-1}	0.0	0.5	-1.4	0.2	-1.1	1.3	0.7
Ec / kJ mol^{-1}	-47.40	-48.70	-47.15	-47.20	-46.94	-46.90	-46.48
ΔEc / kJ mol^{-1}	1.30	0.00	1.55	1.51	1.76	1.80	2.22

[a]Rotational constants (A, B, C). [b]Watson's S-reduction centrifugal distortion constants (D_J, D_{JK}, D_K, d_1, d_2) and electric dipole moments (μ_α, α = a, b, c). [c]Structural data of the S-H···S hydrogen bond. [d]Relative energy with zero-point corrections (ΔE), Gibbs energy (ΔG, 298K, 1 atm) and complexation energy (ΔE_c including BSSE corrections).

Table S1. Continued (2/3).

Isomer	8	9	10	11	12	13	14
	TT-2	CT-4	CT-5	TT-3	CT-6	TT-4	CC-4
A / MHz	397.2	353.3	305.1	355.9	314.0	310.4	357.2
B / MHz	222.4	246.9	250.5	246.2	236.2	248.4	235.4
C / MHz	190.2	193.6	226.4	194.2	233.4	226.2	204.5
D_J / kHz	0.011	-0.005	0.016	-0.003	0.017	0.016	0.009
D_{JK} / kHz	-0.001	0.251	0.010	0.266	0.009	0.022	0.021
D_K / kHz	0.048	-0.207	0.040	-0.212	0.012	0.032	0.030
d_1 / kHz	0.001	0.000	-0.001	-0.001	-0.002	-0.001	0.000
d_2 / kHz	-0.001	-0.004	0.000	-0.005	0.002	0.000	0.001
$\|\mu_a\|$ / D	0.9	1.6	0.3	0.9	0.5	1.1	1.6
$\|\mu_b\|$ / D	1.4	0.2	0.5	1.1	0.5	0.2	0.7
$\|\mu_c\|$ / D	1.4	1.2	0.1	0.3	0.1	0.7	0.7
r(S···H) / Å	2.910	2.821		2.792			2.917
∠(S-H···S) / deg	148.9	135.1		137.4			136.7
centroid-centroid		3.644	3.554	3.633	3.456	3.560	3.545
ΔE_{ZPE} / kJ mol^{-1}	1.5	1.5	1.8	1.8	2.1	2.2	2.2
ΔG / kJ mol^{-1}	0.6	2.8	1.2	3.3	1.0	1.2	3.3
Ec / kJ mol^{-1}	-47.28	-46.40	-46.69	-47.36	-46.44	-46.74	-45.98
ΔEc / kJ mol^{-1}	1.42	2.30	2.01	1.34	2.26	1.97	2.72

[a]Parameter definition as in Table S1.

Table S1. Continued (3/3).

Isomer	15	16	17	18	19
	TT-5	CC-5	CT-7	TT-6	CT-8
A / MHz	399.2	292.5	357.1	383.0	363.2
B / MHz	227.6	268.8	233.5	217.5	240.8
C / MHz	178.5	197.2	201.3	176.0	171.8
D_J / kHz	0.007	0.046	0.029	0.014	0.034
D_{JK} / kHz	0.016	-0.055	-0.022	-0.035	-0.048
D_K / kHz	0.054	0.019	0.140	0.123	0.018
d_1 / kHz	-0.002	-0.006	0.011	-0.005	-0.012
d_2 / kHz	-0.001	0.007	-0.004	-0.001	0.000
$\|\mu_a\|$ / D	1.0	1.2	1.3	0.4	0.4
$\|\mu_b\|$ / D	1.1	0.7	1.4	0.7	1.1
$\|\mu_c\|$ / D	0.4	0.6	0.8	1.7	0.7
r(S···H) / Å	2.826	3.192	2.844		
∠(S-H···S) / deg	146.4	117.2	127.6		
centroid-centroid	3.571	3.513		3.616	3.742
ΔE_{ZPE} / kJ mol^{-1}	2.3	2.3	2.4	3.9	4.3
ΔG / kJ mol^{-1}	3.0	0.5	0.5	4.4	3.6
Ec / kJ mol^{-1}	-45.69	-45.90	-46.19	-	-
Δ*Ec* / kJ mol^{-1}	3.01	2.80	2.51	-	-

[a]Parameter definition as in Table S1.

Table S2. Conformational search and prediction of the rotational and energetic parameters for the 2-naphthalenethiol dimer using the B3LYP-D3(BJ) method and a triple-ζ basis set including polarized and diffuse functions (def2-TZVPD).

Isomer	1 (C_2)	2	3	4	5 (C_2)	6	7
	CC-1	CC-2	CT-1	CT-2	TT-1	CT-3	CC-3
A / MHz [a]	315.7	302.9	292.7	306.1	297.4	389.1	361.3
B / MHz	233.4	250.6	245.9	249.6	242.0	227.9	231.8
C / MHz	231.8	225.5	241.3	225.1	241.3	175.0	197.3
D_J / kHz	0.016	0.011	0.022	0.011	0.028	0.011	0.021
D_{JK} / kHz	0.000	0.030	-0.032	0.034	-0.049	0.003	-0.004
D_K / kHz	0.020	0.005	0.051	0.003	0.073	0.072	0.091
d_1 / kHz	0.002	0.001	-0.006	0.002	0.012	-0.001	0.006
d_2 / kHz	0.001	-0.001	0.001	-0.001	0.001	-0.002	-0.002
$\|\mu_a\|$ / D	0.0	0.2	1.0	-0.7	0.0	-1.2	1.4
$\|\mu_b\|$ / D	0.0	-0.6	1.2	1.3	0.1	1.3	0.7
$\|\mu_c\|$ / D	-1.3	-0.7	0.3	1.3	-2.1	0.1	0.3
r(S···H) / Å						2.838	2.905
∠(S-H···S) / deg						140.6	133.5
centroid-centroid	3.288	3.297	3.299	3.310	3.298	3.328	3.392
ΔE_{ZPE}/ kJ mol^{-1}	0.0	0.0	0.2	0.2	0.0	0.9	0.9
ΔG / kJ mol^{-1}	3.2	3.9	2.5	2.6	0.0	4.2	3.7
Ec / kJ mol^{-1}	-48.55	-47.50	-47.38	-47.25	-47.17	-47.08	-46.70
ΔEc / kJ mol^{-1}	0.0	1.1	1.2	1.3	1.4	1.5	1.8

[a]Parameter definition as in Table S1.

Table S2. Continued (2/3).

Isomer	8	9	10	11	12	13	14
	TT-2	CT-4	CT-5	TT-3	CT-6	TT-4	CC-4
A / MHz	395.3	352.4	306.5	354.5	312.1	308.0	355.7
B / MHz	222.5	246.9	249.4	246.5	236.1	248.6	235.4
C / MHz	189.9	193.3	225.6	193.9	233.5	226.2	204.4
D_J / kHz	0.011	-0.005	0.013	-0.004	0.017	0.014	0.009
D_{JK} / kHz	-0.002	0.244	0.021	0.264	0.011	0.019	0.021
D_K / kHz	0.050	-0.200	0.031	-0.215	0.010	0.036	0.027
d_1 / kHz	0.001	-0.869	0.001	-0.001	0.001	0.001	0.001
d_2 / kHz	-0.012	-0.004	-0.001	-0.005	0.001	0.000	0.001
$\|\mu_a\|$ / D	-1.0	-1.6	-0.3	-1.0	0.5	1.1	-1.6
$\|\mu_b\|$ / D	-1.5	-0.2	-0.5	-1.0	-0.5	-0.2	0.8
$\|\mu_c\|$ / D	-1.4	1.2	0.2	0.3	-0.1	-0.7	-0.6
r(S···H) / Å	2.929	2.827		2.799			2.938
∠(S-H···S) / deg	147.9	134.9		136.9			136.9
centroid-centroid	3.333	3.358	3.320	3.370	3.313	3.334	3.325
ΔE_{ZPE}/ kJ mol^{-1}	1.0	1.3	1.2	1.4	1.6	1.4	2.0
ΔG / kJ mol^{-1}	3.6	6.2	4.3	6.4	3.7	3.4	6.6
Ec / kJ mol^{-1}	-47.38	-46.83	-46.58	-47.46	-46.70	-46.58	-46.12
ΔEc / kJ mol^{-1}	1.2	1.7	2.0	1.1	1.8	2.0	2.4

[a]Parameter definition as in Table S1.

Table S2. Continued (3/3).

Isomer	15	16	17	18	19
	TT-5	CC-5	CT-7	TT-6	CT-8
A / MHz	399.1	291.9	355.9	379.9	363.2
B / MHz	227.0	268.2	233.2	218.2	240.0
C / MHz	178.5	197.0	200.6	175.9	171.5
D_J / kHz	0.008	0.053	0.026	0.015	0.034
D_{JK} / kHz	0.012	-0.065	-0.012	-0.040	-0.048
D_K / kHz	0.062	0.021	0.108	0.131	0.018
d_1 / kHz	-0.002	-0.005	0.008	-0.005	-0.012
d_2 / kHz	0.000	0.010	-0.003	-0.001	-0.002
$\|\mu_a\|$ / D	-1.1	-1.1	-1.4	0.4	0.4
$\|\mu_b\|$ / D	1.1	-0.7	1.4	-0.7	-1.0
$\|\mu_c\|$ / D	0.4	-0.5	0.8	-1.7	-0.7
r(S···H) / Å	2.830	3.276	2.852		
∠(S-H···S) / deg	146.3	113.9	132.9		
centroid-centroid	3.436	3.348	3.394	3.317	3.356
ΔE_{ZPE}/ kJ mol^{-1}	1.9	2.1	1.9	3.9	3.6
ΔG / kJ mol^{-1}	5.5	3.2	3.2	7.5	6.2
Ec / kJ mol^{-1}	-46.33	-45.65	-45.82	-45.82	-43.60
ΔEc / kJ mol^{-1}	2.2	2.9	2.7	2.7	5.0

[a]Parameter definition as in Table S1.

Table S3. Reoptimization of the seven most stable isomers of the 2-naphthalenethiol dimer using the B3LYP-D3(BJ) method and a correlation-consistent polarized (cc-pVTZ) triple-ζ basis set.

Isomer	1 (C_2)	2	3	4	5 (C_2)	6	7
	CC-1	CC-2	CT-1	CT-2	TT-1	CT-3	CC-3
A / MHz [a]	313.4	303.3	294.1	306.3	297.4	390.3	361.3
B / MHz	235.0	250.3	245.9	248.4	241.8	227.1	231.7
C / MHz	233.2	225.8	241.2	226.8	241.4	175.1	197.2
D_J / kHz	0.014	0.010	0.019	0.011	0.024	0.008	0.014
D_{JK} / kHz	-0.001	0.028	-0.020	0.035	-0.033	0.008	0.012
D_K / kHz	0.021	-0.002	0.032	-0.005	0.051	0.052	0.052
d_1 / kHz	-0.001	0.000	-0.005	0.001	-0.009	-0.001	0.003
d_2 / kHz	0.001	-0.001	0.002	-0.001	0.002	-0.001	-0.002
$\|\mu_a\|$ / D	0.0	0.2	1.0	0.8	0.0	-1.1	1.4
$\|\mu_b\|$ / D	1.4	-0.6	1.2	1.2	0.2	1.3	0.8
$\|\mu_c\|$ / D	0.0	-0.7	0.3	1.4	-2.1	0.1	0.3
r(H···S) / Å						2.860	2.902
∠(S-H···S) / deg						139.9	133.6
ΔE_{ZPE}/ kJ mol^{-1}	0.0	0.2	0.3	0.3	0.1	0.8	0.8
ΔG / kJ mol^{-1}	0.9	0.8	0.1	0.0	2.7	1.1	5.3
Ec / kJ mol^{-1}	-48.07	-47.49	-47.24	-47.20	-47.15	-47.24	-46.78
ΔEc / kJ mol^{-1}	0.0	0.6	0.8	0.9	0.9	0.8	1.3

[a]Parameter definition as in Table S1.

Table S4. Reoptimization of the thirteen most stable isomers of the 2-naphthalenethiol dimer using the ωB97XD method and the def2-TZVP basis set.

Isomer	1 (C_2)	2	3	4	5 (C_2)	6	7
	CC-1	CC-2	CT-1	CT-2	TT-1	CT-3	CC-3
A / MHz [a]	314.9	299.1	292.1	303.0	302.9	384.0	360.5
B / MHz	235.6	251.5	245.4	249.5	243.7	229.2	232.6
C / MHz	230.4	226.1	238.6	226.1	231.1	173.5	196.1
D_J / kHz	0.016	0.012	0.019	0.011	0.014	0.007	0.012
D_{JK} / kHz	0.008	0.028	-0.019	0.036	0.007	0.007	0.022
D_K / kHz	0.001	-0.002	0.035	-0.009	0.014	0.040	0.029
d_1 / kHz	-0.002	0.000	-0.002	0.001	0.006	-0.002	0.002
d_2 / kHz	0.002	-0.001	-0.002	-0.001	0.000	-0.001	-0.001
$\|\mu_a\|$ / D	0.0	0.2	0.9	0.7	0.0	1.4	1.6
$\|\mu_b\|$ / D	0.0	0.7	0.6	1.5	0.0	1.4	0.8
$\|\mu_c\|$ / D	1.5	0.8	1.3	1.4	2.4	0.0	0.4
r(S···H) / Å						2.878	2.983
∠(S-H···S) / deg						142.1	133.2
ΔE_{ZPE}/ kJ mol^{-1}	0.7	0.0	0.8	0.4	1.5	1.8	1.0
ΔG / kJ mol^{-1}	0.8	0.1	0.6	0.0	2.1	4.4	1.4
Ec / kJ mol^{-1}	-45.57	-44.39	-43.76	-44.10	-43.72	-43.60	-43.47
ΔEc / kJ mol^{-1}	0.0	1.2	1.8	1.5	1.9	2.0	2.1

[a]Parameter definition as in Table S1.

Table S4. Continued (2/2).

Isomer	8	9	10	11	12	13
	TT-2	CT-4	CT-5	TT-3	CT-6	TT-4
A / MHz	389.5	347.3	302.4	349.3	310.0	305.2
B / MHz	222.8	247.9	250.1	247.5	238.1	248.3
C / MHz	189.9	193.0	225.8	193.7	232.0	226.5
D_J / kHz	0.011	-0.004	0.018	-0.009	0.016	0.014
D_{JK} / kHz	-0.022	0.191	-0.002	0.351	0.018	0.022
D_K / kHz	0.092	-0.158	0.050	-0.302	-0.005	0.024
d_1 / kHz	0.001	0.000	-0.001	0.000	-0.001	0.000
d_2 / kHz	-0.001	-0.004	0.000	-0.007	-0.001	-0.001
$\|\mu_a\|$ / D	1.3	1.9	0.3	1.2	0.5	1.2
$\|\mu_b\|$ / D	1.5	0.2	0.6	1.2	0.6	0.1
$\|\mu_c\|$ / D	1.5	1.2	0.2	0.3	0.3	0.9
r(S···H) / Å	3.065	2.884		2.881		
∠(S-H···S) / deg	106.1	137.7		138.0		
ΔE_{ZPE}/ kJ mol^{-1}	1.3	1.8	1.8	2.4	2.2	2.2
ΔG / kJ mol^{-1}	1.3	3.5	1.1	3.3	0.6	1.4
Ec / kJ mol^{-1}	-43.85	-43.01	-42.84	-43.05	-43.09	-43.22
Δ*Ec* / kJ mol^{-1}	1.7	2.6	2.7	2.5	2.5	2.4

[a]Parameter definition as in Table S1.

Table S5. Reoptimization of the six most stable isomers of the 2-naphthalenethiol dimer using the B2PLYP-D3(BJ) method and the def2-TZVP basis set.

Isomer	1 (C_2)	2	3	4	5 (C_2)	6	7
	CC-1	CC-2	CT-1	CT-2	TT-1	CT-3	CC-3
A / MHz [a]	317.7	305.6	295.4	307.4	301.9	391.5	365.9
B / MHz	233.0	250.8	245.2	250.0	242.3	227.8	231.1
C / MHz	231.9	225.1	240.6	225.8	237.3	175.5	196.7
$\|\mu_a\|$ / D	0.1	0.3	0.4	0.6	0.0	1.2	1.4
$\|\mu_b\|$ / D	0.7	0.6	1.2	1.6	0.0	1.2	0.6
$\|\mu_c\|$ / D	1.1	0.6	0.9	1.0	2.2	0.2	0.3
r(S···H) / Å						2.856	2.937
∠(S-H···S) / deg						140.6	130.5
centroid-centroid	3.460	3.544	3.453	3.541	3.444	3.651	3.635
ΔE / kJ mol^{-1} [b]	0.0	1.4	2.6	1.8	3.3	1.0	1.1
Ec / kJ mol^{-1}	-48.47	-47.25	-46.79	-43.85	-43.30	-46.66	-46.16
ΔEc / kJmol^{-1}	0.0	1.2	1.7	4.6	5.17	1.8	2.3

[a]Parameter definition as in Table S1. [b]Electronic energy uncorrected for zero-point vibrational contributions. The calculation of vibrational frequencies for this method was not possible with our computational resources.

Table S6. Reoptimization of the six most stable isomers of the 2-naphthalenethiol dimer using the RI-SCS-MP2 method and the def2-TZVP basis set.

Isomer	1 (C_2)	2	3	4	5 (C_2)	6	7
	CC-1	CC-2	CT-1	CT-2	TT-1	CT-3	CC-3
A / MHz [a]	315.0	312.4	296.0	314.4	300.6	399.0	375.6
B / MHz	234.5	248.4	244.8	247.4	241.7	220.5	229.0
C / MHz	233.1	224.2	241.1	225.7	239.3	190.9	195.3
$\|\mu_a\|$ / D	0.0	0.2	0.9	0.7	0.0	1.1	1.6
$\|\mu_b\|$ / D	1.5	0.7	1.1	1.4	0.0	1.2	0.6
$\|\mu_c\|$ / D	0.0	0.6	0.8	1.4	2.3	0.1	0.3
r(S···H) / Å						2.856	2.937
∠(S-H···S) / deg						140.6	130.5
ΔE / kJ mol^{-1} [b]	1.1	0.0	0.4	0.4	1.1	1.3	1.9

[a]Parameter definition as in Table S1. [b]Electronic energy uncorrected for zero-point vibrational contributions. The calculation of vibrational frequencies for this method was not possible with our computational resources.

Table S7. Single-point energy calculation using DLPNO-CCSD(T)/def2-TZVP for the B3LYP-D3(BJ) geometries of Table S1.

Isomer	1 (C_2)	2	3	4	5 (C_2)	6	7
	CC-1	CC-2	CT-1	CT-2	TT-1	CT-3	CC-3
A / MHz [a]	318.1	304.2	294.6	307.2	300.3	390.1	363.6
B / MHz	233.4	250.7	245.7	249.6	242.1	228.2	231.9
C / MHz	231.3	225.7	240.9	225.6	239.4	175.3	196.9
$\|\mu_a\|$ / D	0.0	0.20	1.07	0.77	0.00	1.59	1.86
$\|\mu_b\|$ / D	0.0	0.81	1.47	1.62	0.01	1.45	0.85
$\|\mu_c\|$ / D	1.63	0.88	0.73	1.51	2.59	0.08	0.38
ΔE / kJ mol^{-1} [b]	0.69	0.00	0.61	0.76	1.07	2.40	1.40

[a]Parameter definition as in Table S1. [b]Electronic energy uncorrected for zero-point vibrational contributions. Neither geometry optimizations nor vibrational frequency calculations were possible for this method with our computational resources.

Table S8. Observed rotational transitions of isomer 1 (CC-1) of the 2-naphtalenethiol dimer (Freq.), together with the differences with the calculated transitions (o.-c.) for the fit of Table 1 and estimated frequency uncertainties (unc.). All values in MHz.

N	*J'*	K_{-1}'	K_{+1}'	*J"*	K_{-1}''	K_{+1}''	*Freq.* / MHz	*o-c* / MHz	*unc.* / MHz
1	4	2	2	3	1	2	2060.0280	-0.0110	0.0200
2	4	3	1	3	2	1	2229.2138	0.0009	0.0200
3	4	3	2	3	2	2	2230.3296	0.0004	0.0200
4	4	4	0	3	3	0	2388.0131	0.0020	0.0200
5	4	4	1	3	3	1	2388.0131	-0.0046	0.0200
6	5	1	4	4	0	4	2410.7488	-0.0154	0.0200
7	5	2	3	4	1	3	2513.1333	0.0039	0.0200
8	5	2	4	4	1	4	2554.7442	-0.0016	0.0200
9	6	1	5	5	2	3	2554.8645	-0.0172	0.0200
10	5	3	2	4	2	2	2686.4994	0.0020	0.0200
11	5	3	3	4	2	3	2689.7849	0.0103	0.0200
12	5	4	1	4	3	1	2846.6171	0.0433	0.0200
13	5	4	2	4	3	2	2846.6171	-0.0031	0.0200
14	6	1	5	5	0	5	2886.6407	-0.0053	0.0200
15	5	5	0	4	4	0	3004.7874	0.0033	0.0200
16	5	5	1	4	4	1	3004.7874	0.0031	0.0200
17	7	1	6	6	2	4	3020.6630	-0.0128	0.0200
18	6	2	5	5	1	5	3025.5780	-0.0129	0.0200
19	6	3	3	5	2	3	3142.5046	-0.0004	0.0200
20	6	3	4	5	2	4	3149.9295	0.0074	0.0200
21	6	4	2	5	3	2	3305.0160	-0.0079	0.0200
22	6	4	3	5	3	3	3305.2031	-0.0052	0.0200
23	7	1	6	6	0	6	3366.6879	-0.0148	0.0200
24	7	2	5	6	1	5	3422.4833	0.0074	0.0200
25	6	5	1	5	4	1	3463.3759	-0.0011	0.0200
26	6	5	2	5	4	2	3463.3759	-0.0026	0.0200
27	8	1	7	7	2	5	3483.6740	-0.0014	0.0200
28	7	2	6	6	1	6	3498.8873	-0.0010	0.0200
29	7	3	4	6	2	4	3596.8148	0.0049	0.0200
30	7	3	5	6	2	5	3611.0329	0.0028	0.0200
31	6	6	0	5	5	0	3621.5466	-0.0018	0.0200
32	6	6	1	5	5	1	3621.5466	-0.0018	0.0200
33	7	4	3	6	3	3	3763.2485	0.0043	0.0200
34	7	4	4	6	3	4	3763.7872	-0.0023	0.0200
35	8	2	6	7	1	6	3880.6241	0.0095	0.0200
36	9	0	9	8	1	7	3905.8943	0.0162	0.0200
37	7	5	2	6	4	2	3921.9396	0.0046	0.0200
38	7	5	3	6	4	3	3921.9396	-0.0026	0.0200
39	9	1	8	8	2	6	3942.7230	-0.0033	0.0200
40	8	2	7	7	1	7	3974.5965	-0.0181	0.0200
41	8	3	5	7	2	5	4049.1667	-0.0070	0.0200

42	8	3	6	7	2	6	4073.3657	0.0004	0.0200
43	7	6	2	6	5	2	4080.1486	0.0038	0.0200
44	7	6	1	6	5	1	4080.1486	0.0038	0.0200
45	8	4	4	7	3	4	4221.0606	0.0027	0.0200
46	8	4	5	7	3	5	4222.4007	0.0026	0.0200
47	7	7	0	6	6	0	4238.3104	0.0037	0.0200
48	7	7	1	6	6	1	4238.3104	0.0037	0.0200
49	9	1	8	8	0	8	4340.1041	-0.0147	0.0200
50	9	2	7	8	1	7	4342.0986	0.0100	0.0200
51	8	5	3	7	4	3	4380.4293	0.0022	0.0200
52	8	5	4	7	4	4	4380.4293	-0.0242	0.0200
53	10	1	9	9	2	7	4396.8112	-0.0148	0.0200
54	9	2	8	8	1	8	4452.6986	-0.0182	0.0200
55	9	3	6	8	2	6	4499.6763	-0.0047	0.0200
56	9	3	7	8	2	7	4537.2000	0.0121	0.0200
57	8	6	2	7	5	2	4538.7244	0.0045	0.0200
58	8	6	3	7	5	3	4538.7244	0.0042	0.0200
59	9	4	5	8	3	5	4678.2238	0.0045	0.0200
60	9	4	6	8	3	6	4681.1106	0.0069	0.0200
61	8	7	1	7	6	1	4696.9002	-0.0024	0.0200
62	8	7	2	7	6	2	4696.9002	-0.0024	0.0200
63	11	2	9	10	3	7	4725.2741	0.0015	0.0200
64	10	2	8	9	1	8	4807.4815	0.0045	0.0200
65	9	5	4	8	4	4	4838.8544	0.0433	0.0200
66	9	5	5	8	4	5	4838.8544	-0.0353	0.0200
67	11	1	10	10	2	8	4845.1377	-0.0156	0.0200
68	8	8	0	7	7	0	4855.0609	0.0026	0.0200
69	8	8	1	7	7	1	4855.0609	0.0026	0.0200
70	10	3	7	9	2	7	4948.8127	0.0054	0.0200
71	9	6	3	8	5	3	4997.2588	0.0004	0.0200
72	9	6	4	8	5	4	4997.2588	-0.0006	0.0200
73	10	3	8	9	2	8	5002.7399	0.0000	0.0200
74	10	4	6	9	3	6	5134.4067	-0.0026	0.0200
75	10	4	7	9	3	7	5140.0217	0.0036	0.0200
76	9	7	3	8	6	3	5155.4812	-0.0019	0.0200
77	9	7	2	8	6	2	5155.4812	-0.0019	0.0200
78	13	4	10	12	5	8	5259.5131	0.0013	0.0200
79	13	4	9	12	5	7	5263.2800	-0.0192	0.0200
80	11	2	9	10	1	9	5277.2762	0.0223	0.0200
81	10	5	5	9	4	5	5297.0301	0.0017	0.0200
82	10	5	6	9	4	6	5297.2345	0.0038	0.0200
83	9	8	2	8	7	2	5313.6506	-0.0016	0.0200
84	9	8	1	8	7	1	5313.6506	-0.0016	0.0200
85	11	3	8	10	2	8	5397.3976	0.0073	0.0200
86	10	6	4	9	5	4	5455.7409	-0.0002	0.0200
87	10	6	5	9	5	5	5455.7409	-0.0037	0.0200
88	11	3	9	10	2	9	5470.2354	0.0019	0.0200
89	9	9	1	8	8	1	5471.8025	0.0004	0.0200
90	9	9	0	8	8	0	5471.8025	0.0004	0.0200

91	11	4	7	10	3	7	5589.2326	-0.0175	0.0200
92	11	4	8	10	3	8	5599.3095	0.0121	0.0200
93	10	7	3	9	6	3	5614.0394	0.0011	0.0200
94	10	7	4	9	6	4	5614.0394	0.0011	0.0200
95	12	2	10	11	1	10	5751.8471	0.0291	0.0200
96	11	5	6	10	4	6	5755.0043	0.0066	0.0200
97	11	5	7	10	4	7	5755.4673	0.0045	0.0200
98	10	8	3	9	7	3	5772.2417	0.0077	0.0200
99	10	8	2	9	7	2	5772.2417	0.0077	0.0200
100	12	3	9	11	2	9	5846.5104	-0.0068	0.0200
101	14	1	14	13	2	12	5898.4714	0.0174	0.0200
102	11	6	5	10	5	5	5914.1583	0.0133	0.0200
103	11	6	6	10	5	6	5914.1583	0.0027	0.0200
104	10	9	2	9	8	2	5930.3935	0.0002	0.0200
105	10	9	1	9	8	1	5930.3935	0.0002	0.0200
106	12	4	8	11	3	8	6042.3360	-0.0105	0.0200
107	12	4	9	11	3	9	6059.1487	0.0092	0.0200
108	11	7	4	10	6	4	6072.5552	-0.0006	0.0200
109	11	7	5	10	6	5	6072.5552	-0.0008	0.0200
110	10	10	1	9	9	1	6088.5405	0.0031	0.0200
111	10	10	0	9	9	0	6088.5405	0.0031	0.0200
112	12	5	7	11	4	7	6212.6100	0.0032	0.0200
113	12	5	8	11	4	8	6213.5804	-0.0052	0.0200
114	11	8	4	10	7	4	6230.7958	-0.0002	0.0200
115	11	8	3	10	7	3	6230.7958	-0.0002	0.0200
116	13	2	11	12	1	11	6231.5065	0.0345	0.0200
117	13	3	10	12	2	10	6297.3809	0.0119	0.0200
118	12	6	6	11	5	6	6372.4279	-0.0134	0.0200
119	12	6	7	11	5	7	6372.4279	-0.0413	0.0200
120	11	9	3	10	8	3	6388.9739	0.0001	0.0200
121	11	9	2	10	8	2	6388.9739	0.0001	0.0200
122	13	4	9	12	3	9	6493.3568	-0.0094	0.0200
123	13	4	10	12	3	10	6519.7949	0.0183	0.0200
124	12	7	5	11	6	5	6531.0237	0.0016	0.0200
125	12	7	6	11	6	6	6531.0237	0.0012	0.0200
126	11	10	2	10	9	2	6547.1238	-0.0011	0.0200
127	11	10	1	10	9	1	6547.1238	-0.0011	0.0200
128	13	5	8	12	4	8	6669.6943	-0.0081	0.0200
129	13	5	9	12	4	9	6671.6148	-0.0038	0.0200
130	12	8	5	11	7	5	6689.3300	-0.0004	0.0200
131	12	8	4	11	7	4	6689.3300	-0.0004	0.0200
132	11	11	1	10	10	1	6705.2644	0.0014	0.0200
133	11	11	0	10	10	0	6705.2644	0.0014	0.0200
134	14	3	11	13	2	11	6751.0938	0.0234	0.0200
135	13	6	7	12	5	7	6830.6078	0.0124	0.0200
136	13	6	8	12	5	8	6830.6078	-0.0545	0.0200
137	12	9	3	11	8	3	6847.5395	0.0011	0.0200
138	12	9	4	11	8	4	6847.5395	0.0011	0.0200
139	14	4	10	13	3	10	6942.1322	-0.0183	0.0200

140	14	4	11	13	3	11	6981.4811	0.0171	0.0200
141	13	7	6	12	6	6	6989.4210	0.0001	0.0200
142	13	7	7	12	6	7	6989.4210	-0.0012	0.0200
143	12	10	3	11	9	3	7005.7020	-0.0008	0.0200
144	12	10	2	11	9	2	7005.7020	-0.0008	0.0200
145	14	5	9	13	4	9	7126.0865	0.0056	0.0200
146	14	5	10	13	4	10	7129.5957	-0.0121	0.0200
147	13	8	6	12	7	6	7147.8257	-0.0014	0.0200
148	13	8	5	12	7	5	7147.8257	-0.0014	0.0200
149	12	11	2	11	10	2	7163.8471	0.0008	0.0200
150	12	11	1	11	10	1	7163.8471	0.0008	0.0200
151	14	6	8	13	5	8	7288.5752	0.0121	0.0200
152	14	6	9	13	5	9	7288.6842	-0.0274	0.0200
153	13	9	5	12	8	5	7306.0786	-0.0019	0.0200
154	13	9	4	12	8	4	7306.0786	-0.0019	0.0200
155	12	12	1	11	11	1	7321.9780	0.0000	0.0200
156	12	12	0	11	11	0	7321.9780	0.0000	0.0200
157	15	3	13	14	2	13	7362.4045	-0.0071	0.0200
158	15	4	12	14	3	12	7444.4919	0.0251	0.0200
159	13	10	3	12	9	3	7464.2613	-0.0056	0.0200
160	13	10	4	12	9	4	7464.2613	-0.0056	0.0200
161	15	5	10	14	4	10	7581.4677	-0.0114	0.0200
162	15	6	9	14	5	9	7746.2617	-0.0260	0.0200
163	15	6	10	14	5	10	7746.5936	-0.0032	0.0200
164	14	9	6	13	8	6	7764.5976	0.0039	0.0200
165	14	9	5	13	8	5	7764.5976	0.0039	0.0200
166	13	12	2	12	11	2	7780.5533	-0.0033	0.0200
167	13	12	1	12	11	1	7780.5533	-0.0033	0.0200
168	15	7	8	14	6	8	7905.9384	-0.0016	0.0200
169	15	7	9	14	6	9	7905.9384	-0.0106	0.0200
170	14	10	5	13	9	5	7922.8183	0.0059	0.0200
171	14	10	4	13	9	4	7922.8183	0.0059	0.0200
172	13	13	1	12	12	1	7938.6854	0.0039	0.0200
173	13	13	0	12	12	0	7938.6854	0.0039	0.0200

Table S9. Observed rotational transitions of isomer 2 (CC2) of the 2-naphtalenethiol dimer (Freq.), together with the differences with the calculated transitions (o.-c.) for the fit of Table 1 and estimated frequency uncertainties (unc.). All values in MHz.

N	*J'*	K_{-1}'	K_{+1}'	*J''*	K_{-1}''	K_{+1}''	*Freq.* / MHz	*o-c* / MHz	*unc.* / MHz
1	5	5	1	4	4	1	2930.6875	0.0274	0.0200
2	5	5	0	4	4	1	2930.6875	-0.0011	0.0200
3	6	6	1	5	5	0	3529.4887	0.0115	0.0200
4	6	6	0	5	5	0	3529.4887	0.0078	0.0200
5	6	6	1	5	5	1	3529.4887	-0.0170	0.0200
6	6	6	0	5	5	1	3529.4887	-0.0208	0.0200
7	7	7	1	6	6	0	4128.3980	0.0029	0.0200
8	7	7	0	6	6	0	4128.3980	0.0025	0.0200
9	7	7	1	6	6	1	4128.3980	-0.0007	0.0200
10	7	7	0	6	6	1	4128.3980	-0.0012	0.0200
11	10	0	10	9	1	9	4475.3000	0.0277	0.0200
12	10	1	10	9	0	9	4475.3000	-0.0285	0.0200
13	8	7	2	7	6	1	4599.2000	-0.0209	0.0200
14	8	7	1	7	6	1	4599.2000	-0.0278	0.0200
15	8	7	2	7	6	2	4599.2875	0.0191	0.0200
16	8	7	1	7	6	2	4599.2875	0.0123	0.0200
17	9	2	7	8	1	7	4726.8375	-0.0078	0.0200
18	8	8	1	7	7	0	4727.2813	-0.0060	0.0200
19	8	8	0	7	7	0	4727.2813	-0.0060	0.0200
20	8	8	1	7	7	1	4727.2813	-0.0065	0.0200
21	8	8	0	7	7	1	4727.2813	-0.0065	0.0200
22	9	8	2	8	7	1	5198.2000	0.0168	0.0200
23	9	8	1	8	7	1	5198.2000	0.0159	0.0200
24	9	8	2	8	7	2	5198.2000	0.0100	0.0200
25	9	8	1	8	7	2	5198.2000	0.0091	0.0200
26	9	9	1	8	8	0	5326.1705	0.0046	0.0200
27	9	9	0	8	8	0	5326.1705	0.0046	0.0200
28	9	9	1	8	8	1	5326.1705	0.0046	0.0200
29	9	9	0	8	8	1	5326.1705	0.0046	0.0200
30	10	9	2	9	8	1	5797.1000	0.0063	0.0200
31	10	9	1	9	8	1	5797.1000	0.0062	0.0200
32	10	9	2	9	8	2	5797.1000	0.0054	0.0200
33	10	9	1	9	8	2	5797.1000	0.0053	0.0200
34	11	5	7	10	4	7	5810.2625	0.0107	0.0200
35	10	10	1	9	9	0	5925.0375	0.0059	0.0200
36	10	10	0	9	9	0	5925.0375	0.0059	0.0200
37	10	10	1	9	9	1	5925.0375	0.0059	0.0200
38	10	10	0	9	9	1	5925.0375	0.0059	0.0200
39	11	10	2	10	9	1	6395.9875	0.0094	0.0200
40	11	10	1	10	9	1	6395.9875	0.0093	0.0200
41	11	10	2	10	9	2	6395.9875	0.0092	0.0200
42	11	10	1	10	9	2	6395.9875	0.0092	0.0200
43	12	10	2	11	9	2	6866.7375	0.0019	0.0200
44	12	10	2	11	9	3	6866.7375	0.0007	0.0200

45	12	11	2	11	10	1	6994.8500	0.0074	0.0200
46	12	11	1	11	10	1	6994.8500	0.0074	0.0200
47	12	11	2	11	10	2	6994.8500	0.0074	0.0200
48	12	11	1	11	10	2	6994.8500	0.0074	0.0200
49	12	12	1	11	11	1	7122.7087	-0.0134	0.0200
50	12	12	1	11	11	0	7122.7087	-0.0134	0.0200
51	12	12	0	11	11	1	7122.7087	-0.0134	0.0200
52	12	12	0	11	11	0	7122.7087	-0.0134	0.0200
53	13	11	3	12	10	2	7465.6375	-0.0070	0.0200
54	13	11	2	12	10	2	7465.6375	-0.0070	0.0200
55	13	11	3	12	10	3	7465.6375	-0.0071	0.0200
56	13	11	2	12	10	3	7465.6375	-0.0072	0.0200
57	13	12	2	12	11	1	7593.7125	0.0239	0.0200
58	13	12	1	12	11	1	7593.7125	0.0239	0.0200
59	13	12	2	12	11	2	7593.7125	0.0239	0.0200
60	13	12	1	12	11	2	7593.7125	0.0239	0.0200
61	7	3	4	6	2	4	3606.3517	0.0023	0.0200
62	7	1	6	6	0	6	3697.6256	0.0086	0.0200
63	7	5	3	6	4	3	3873.9339	0.0090	0.0200
64	9	5	4	8	4	5	4850.1746	-0.0103	0.0200
65	9	7	3	8	6	3	5069.8150	0.0175	0.0200
66	9	7	2	8	6	3	5069.8150	-0.0355	0.0200
67	10	8	2	9	7	3	5668.7911	-0.0151	0.0200
68	10	8	3	9	7	3	5668.7911	-0.0069	0.0200
69	13	0	13	12	1	12	5803.7478	0.0008	0.0200
70	13	1	13	12	0	12	5803.7478	-0.0009	0.0200
71	11	9	3	10	8	2	6267.7667	-0.0146	0.0200
72	11	9	2	10	8	2	6267.7667	-0.0158	0.0200
73	11	9	3	10	8	3	6267.7667	-0.0228	0.0200
74	11	9	2	10	8	3	6267.7667	-0.0240	0.0200
75	11	11	1	10	10	1	6523.8767	-0.0073	0.0200
76	11	11	1	10	10	0	6523.8767	-0.0073	0.0200
77	11	11	0	10	10	1	6523.8767	-0.0073	0.0200
78	11	11	0	10	10	0	6523.8767	-0.0073	0.0200
79	12	10	3	11	9	2	6866.7338	-0.0015	0.0200
80	12	10	2	11	9	2	6866.7338	-0.0017	0.0200
81	12	10	3	11	9	3	6866.7338	-0.0027	0.0200
82	12	10	2	11	9	3	6866.7338	-0.0029	0.0200
83	13	13	1	12	12	0	7721.5395	-0.0053	0.0200
84	13	13	0	12	12	1	7721.5395	-0.0053	0.0200
85	13	13	0	12	12	0	7721.5395	-0.0053	0.0200
86	13	13	1	12	12	1	7721.5395	-0.0053	0.0200

Table S10. Equilibrium coordinates for isomer CC-1 of the 2-naphthalenethiol dimer, according to the prediction with B2PLYP-D3(BJ) /def2TZVP.

Atom	*a* / Å[a]	*b* / Å	*c* / Å
C	-3.328760	-0.691931	0.372929
C	-1.298683	-1.171262	2.230257
C	-2.526019	-0.724724	2.646597
C	-3.551870	-0.477980	1.709493
H	-4.113068	-0.509591	-0.351621
H	-0.507800	-1.353574	2.946621
H	-2.714308	-0.561215	3.700049
H	-4.517424	-0.128246	2.051328
C	-2.071912	-1.143375	-0.088008
C	-1.031847	-1.380833	0.856702
C	0.243801	-1.776644	0.397634
C	0.491809	-1.921438	-0.944219
C	-0.548309	-1.706446	-1.882785
C	-1.793077	-1.334485	-1.461683
H	1.032329	-1.926243	1.123721
H	-0.341020	-1.816325	-2.939403
H	-2.575628	-1.146410	-2.185362
S	2.078306	-2.354360	-1.594646
H	2.772205	-2.143202	-0.465555
C	3.328940	0.689303	0.379254
C	1.298313	1.150674	2.240520
C	2.525688	0.700625	2.652947
C	3.551821	0.462941	1.713813
H	4.113486	0.514008	-0.346778
H	0.507226	1.326083	2.958379
H	2.713792	0.527351	3.704870
H	4.517414	0.110348	2.052584
C	2.072034	1.144581	-0.077736
C	1.031699	1.372933	0.868917
C	-0.243984	1.772592	0.413282
C	-0.491762	1.929784	-0.927217
C	0.548592	1.723730	-1.867532
C	1.793387	1.348262	-1.449646
H	-1.032716	1.915147	1.140563
H	0.341500	1.843370	-2.923131
H	2.576155	1.167201	-2.174882
S	-2.078256	2.368043	-1.574010
H	-2.772115	2.148387	-0.446509

[a]Principal inertial axes denoted *a*, *b*, *c*.

Table S11. Equilibrium coordinates for isomer CC-2 of the 2-naphthalenethiol dimer, according to the prediction with B2PLYP-D3(BJ) /def2TZVP.

Atom	*a* / Å[a]	*b* / Å	*c* / Å
C	1.638657	2.672243	1.028461
C	2.509408	0.960173	-0.999093
C	3.404419	1.481793	-0.101716
C	2.966174	2.343529	0.926283
H	1.296399	3.336165	1.813189
H	2.842678	0.285160	-1.776642
H	4.455643	1.234489	-0.180236
H	3.682928	2.748307	1.629006
C	0.690787	2.149111	0.120806
C	1.131016	1.266977	-0.908427
C	0.180803	0.700374	-1.786891
C	-1.154582	0.988695	-1.655981
C	-1.590872	1.881152	-0.645926
C	-0.689877	2.443127	0.212343
H	0.526105	0.011574	-2.546914
H	-2.646131	2.096726	-0.545351
H	-1.032871	3.107417	0.995089
S	-2.411567	0.278930	-2.676368
H	-1.602094	-0.495671	-3.414400
C	-2.856715	-0.935615	1.071738
C	-0.720258	0.057939	2.572042
C	-2.003770	0.445197	2.854241
C	-3.083324	-0.050858	2.094222
H	-3.679692	-1.314356	0.478606
H	0.111524	0.452560	3.142051
H	-2.193608	1.143205	3.659684
H	-4.091465	0.270627	2.321577
C	-1.546008	-1.362908	0.763442
C	-0.453015	-0.850926	1.521395
C	0.862489	-1.248577	1.195281
C	1.095573	-2.119558	0.160541
C	0.008634	-2.640940	-0.583250
C	-1.273161	-2.271683	-0.284852
H	1.684918	-0.832167	1.762463
H	0.202565	-3.325674	-1.399234
H	-2.098897	-2.658947	-0.867617
S	2.716386	-2.633072	-0.331661
H	3.393078	-1.737361	0.402920

[a]Principal inertial axes denoted *a*, *b*, *c*.

Table S12. Rotational constants of the observed dimers of 2-naphtalenethiol compared to four theoretical predictions with B3LYP-D3(BJ), ωB97XD, B2PLYPD3(BJ) and RI-SCS-MP2 using def2-TZVP as basis set, and their relative percentage differences.

**Isomer 1**	Experiment	B3LYP-D3(BJ)	ωB97XD	B2PLYP-D3(BJ)	RI-SCS-MP2
A / MHz	308.38853(21)[a]	318.1[3.1%][b]	314.9[2.1%]	317.7[3.0%]	315.0[2.1%]
B / MHz	231.75029(16)	233.4[0.7%]	235.6[1.7%]	233.0[0.5%]	234.5[1.2%]
C / MHz	226.78483(18)	231.3[2.0%]	230.4[1.6%]	231.9[2.3%]	233.1[2.8%]
B-C / MHz	5.0	2.1	5.2	1.1	1.4
B+C / MHz	458.5	464.7	466	464.8	467.6

**Isomer 2**	Experiment	B3LYP-D3(BJ)	ωB97XD	B2PLYP-D3(BJ)	RI-SCS-MP2
A / MHz	299.45856(51)	304.2[1.6%]	299.1[0.1%]	305.6[2.0%]	312.4[4.3%]
B / MHz	246.9652(12)	250.7[1.5%]	251.5[1.9%]	250.8[1.6%]	248.4[0.6%]
C / MHz	221.5793(15)	225.7[1.9%]	226.1[2.0%]	225.1[1.6%]	224.2[1.2%]
B-C / MHz	25.4	24.9	25.5	25.7	24.2
B+C / MHz	468.5	476.4	477.6	475.9	472.6

[a]Standard errors in parentheses in units of the last digit. [b]Relative percentage differences in square brackets, calculated as (theory − experiment) ⁄ experiment.

Table S13. Rotational constants of the observed dimers of 2-naphtalenethiol compared to the theoretical model B3LYP-D3(BJ) using either the def2-TZVP, def2-TZVPD or cc-pVTZ basis sets, and their relative percentage differences.

**Isomer 1**	Experiment	def2-TZVP	def2-TZVPD	cc-pVTZ
A / MHz	308.38853(21)[a]	318.1[3.1%][b]	315.7[2.4%]	313.4[1.6%]
B / MHz	231.75029(16)	233.4[0.7%]	233.4[0.7%]	235.0[1.4%]
C / MHz	226.78483(18)	231.3[2.0%]	231.8[2.2%]	233.2[2.8%]
B-C / MHz	5.0	2.1	1.6	1.8
B+C / MHz	458.5	464.7	465.2	468.2

**Isomer 2**	Experiment	def2-TZVP	def2-TZVPD	cc-pVTZ
A / MHz	299.45856(51)	304.2[1.6%]	302.9[1.1%]	303.3[1.3%]
B / MHz	246.9652(12)	250.7[1.5%]	250.6[1.5%]	250.3[1.4%]
C / MHz	221.5793(15)	225.7[1.9%]	225.5[1.8%]	225.8[1.9%]
B-C / MHz	25.4	24.9	25.1	24.5
B+C / MHz	468.5	476.4	476.1	476.1

[a]Standard errors in parentheses in units of the last digit. [b]Relative percentage differences in square brackets, calculated as (theory − experiment) ⁄ experiment.
